# Chemical Investigation of the Mediterranean Sponge *Crambe crambe* by UHPLC-HRMS/MS via Manual and Computational Dereplication Approaches

**DOI:** 10.3390/md22110522

**Published:** 2024-11-20

**Authors:** Pinelopi Vlachou, Nikolaos Tsafantakis, Nikola Milic, Alexandros Polyzois, Eirini Baira, Aikaterini Termentzi, Géraldine Le Goff, Jamal Ouazzani, Nikolas Fokialakis

**Affiliations:** 1 Laboratory of Pharmacognosy & Natural Products Chemistry, Department of Pharmacy, National and Kapodistrian University of Athens, 15771 Athens, Greecenmilic@pharm.uoa.gr (N.M.); a.polyzois92@gmail.com (A.P.); e.baira@bpi.gr (E.B.); 2 Laboratory of Toxicological Control of Pesticides, Scientific Directorate of Pesticides’ Control & Phytopharmacy, Benaki Phytopathological Institute, 14561 Kifissia, Greece; a.termentzi@bpi.gr; 3 Institut de Chimie des Substances Naturelles ICSN, Centre National de la Recherche Scientifique, 91198 Gif-sur-Yvette, France; geraldine.legoff@cnrs.fr (G.L.G.); jamal.ouazzani@cnrs.fr (J.O.)

**Keywords:** *Crambe crambe*, UHPLC-HRMS/MS, dereplication, computational mass spectrometry, molecular networking, guanidine alkaloids, crambescin analogues

## Abstract

The CH_2_Cl_2_-MeOH extract of the Mediterranean sponge *Crambe crambe* was investigated via UHPLC-HRMS/MS employing manual dereplication and in silico mass spectrometry tools. A deconvolution approach was implemented for the extensive metabolic characterization of the sample, resulting in the annotation of 53 compounds. The analysis of data-dependent HRMS/MS scans was conducted to establish fragmentation patterns characteristic of each crambescin A, B, and C sub-families. Among the 39 compounds identified from these groups, 22 analogues were reported for the first time including 4 new homologous series that differed by the ratio of methylene units in the upper (*n* + 2) and lower (*m* + 2) alkyl side chains. More specifically, crambescins presenting *m* = 5 or 6 and *n* = 5 (compounds **7**, **11**, **22** and **24**) as well as *m* = 5 or 6 and *n* = 4 (compounds **5**, **6**, **8**, **9**, 12 and 14) were characterized. Additionally, four new features, potentially corresponding to new crambescidin analogues (compounds **13**, **15**, **35**, and **39**), were also reported. The identity of the dereplicated features was further validated by studying crambescins’ spectral similarities through a feature-based molecular networking approach. Overall, this study suggests UHPLC-HRMS/MS—through the integration of manual and computational dereplication approaches—as a valuable tool for the investigation and high-throughput characterization of the *C. crambe* metabolome.

## 1. Introduction

*Crambe crambe* is a red encrusting sponge of the order Poecilosclerida and is widespread along the sublittoral of the western Mediterranean Sea and the Macaronesian archipelagos [1]. Extensive studies have been carried out on this model sponge with regard to its ecology and chemical content [2,3,4].

The species is well known for the accumulation of a large diversity of guanidine-bearing alkaloids, a group of secondary metabolites that might have a microbial origin [5,6]. Although for many years it was believed that *C. crambe* is virtually free of microsymbionts [7], recent studies of the microbiome of the species have reported the presence of a low number of bacteria and fungi, along with a group of Betaproteobacteria which dominates its microbial community and may be involved in the biosynthesis of guanidine alkaloids [6,8,9]. However, *C. crambe* is a low microbial abundance sponge harbouring much smaller bacterial communities with a lower bacterial diversity compared to other sponges [10].

**Figure 1:**
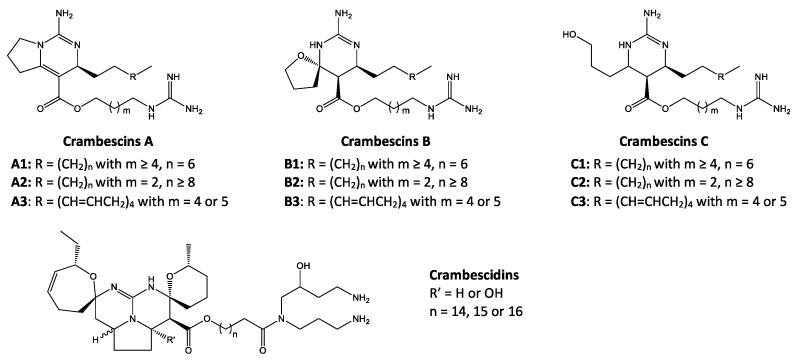
Structures of crambescin sub-families and the most common crambescidins described in the literature.

The complex guanidine alkaloids derived from the sponge are distributed in two main chemical families, crambescins and crambescidins. The crambescins can be divided into three sub-families: crambescins A, B, and C. The structure of all crambescin A analogues includes a 5,6-fused bicyclic ring system linked to one aliphatic side chain at the C-13 position and a guanidinoalkyl side chain at the C-7 position. The second type is of a crambescin B skeleton, which is characterized by a [4,5] decane spiro-ring system with similar side chains as crambescin A. Crambescin C represents the third sub-family featured by a monocyclic guanidine ring connected to a linear 3-hydroxypropyl chain and two side chains [11,12] (Figure 1). The members of the crambescidin family have a pentacyclic guanidine core and are named according to their molecular weight [13,14,15].

These specialized metabolites biosynthesized in *C. crambe* are chemotaxonomic markers for the Crambeidae family and exhibit an outstanding range of biological activities. Several studies have demonstrated the antifungal, antimalarial, antiviral, and cytotoxic activity against a diverse panel of human tumour cell lines from members of the crambescidin family, while crambescins present a more specific activity on ion channels [14,16,17,18,19,20,21,22,23]. Being one of the strongest organic bases, guanidine might be responsible for the biological activity of these molecules, due to the ionic pocket in the guanidine nucleus, which can interact with biopolymers through hydrogen bonds and/or electrostatic interactions [24]. The unique chemical architectures of polycyclic guanidine alkaloids, along with the broad spectrum of their biological activities, make them particularly attractive as starting points for drug development.

The complexity of the *C. crambe* metabolic profile lies in the presence of multiple crambescin homologous series constituting a mixture of compounds that display a high degree of structural similarity. In that context, UHPLC-HRMS/MS associated with the dereplication approach represents a valuable tool for the high-throughput characterization of the sponge’s metabolome. Molecular networking and computational processing contribute to the advanced analysis of MS/MS data. Molecular networking was first introduced as a dereplication strategy over a decade ago, with a key publication in 2013 demonstrating its ability to organize mass spectrometry data and facilitate the identification of known compounds in complex biological mixtures based on spectral similarities [25]. Since then, the approach has evolved considerably, with various advancements and applications reported in the literature [26,27]. Regarding sea sponges, molecular networking as a dereplication strategy has been successfully employed across different species, sometimes leading to the discovery of novel compounds, and even entirely new compound classes [28,29].

In the present study, the dichloromethane–methanol (CH_2_Cl_2_-MeOH) extract of *C. crambe* was analyzed using UHPLC-HRMS/MS. The robust mass spectral data were processed and dereplicated with modern mass spectrometry computational tools that utilize machine learning algorithms and in silico fragmentation to aid in structural elucidation and chemical class prediction [30,31,32,33,34]. Molecular networking visualization was conducted in the comprehensive Cytoscape environment [35] by integrating data from both molecular networking and manual dereplication approaches.

## 2. Results and Discussion

Through this research, the guanidine alkaloid class of natural products was annotated, resulting in the manual dereplication of 53 compounds (Supplementary Material, Table S11). Among these, 22 novel crambescin analogues were reported for the first time (Table 1), along with 4 potentially new crambescidin analogues for which full structural characterization has not yet been achieved.

**Figure 2:**
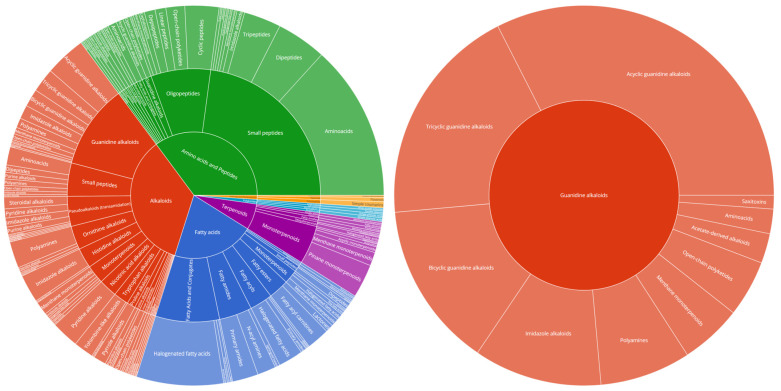
Sunburst plot visualization of relative quantification of Natural Product Classes (NPCs), as predicted by CANOPUS ClassyFire, of the total chemical profile’s NPCs (**left chart**) and subclasses of guanidine alkaloids (**right chart**).

All detected compounds were more abundant both in number and intensity in the ESI(+) mode in comparison to the ESI(−). Consequently, we focused our study only on the ESI(+). The deconvolution process yielded the detection of 53 compounds within the crude extract as presented in Table 2. The two internal standards, yohimbine and reserpine, were detected as intense peaks eluted at 9.80 and 12.59 min, organizing into their own distinct molecular clusters in the MN analysis. The observed *m*/*z* corresponded to [M+H]^+^ ions and were consistent with their molecular formulas, while characteristic fragments ions were detected with a mass error < 5 ppm. These results demonstrated the suitability of the system regarding mass accuracy at both HRMS and HRMS/MS levels, and a mass error of ±5 ppm was further considered for the annotation of deconvoluted peaks. All 53 annotated metabolites belonged to the guanidine alkaloid family, most of them being detected as multiple charged species. Annotation of crambescin analogues was performed according to previous studies suggesting a conventional nomenclature based on sub-family type followed by the molecular weight [11,36]. Accordingly, crambescidin analogues were named according to their molecular weight. Crambescins and, generally, guanidine alkaloids, constitute a major component of the overall chemical profile as demonstrated by the sunburst plots visualizing the LC-MS/MS quantification data (Figure 2).

**Figure 3:**
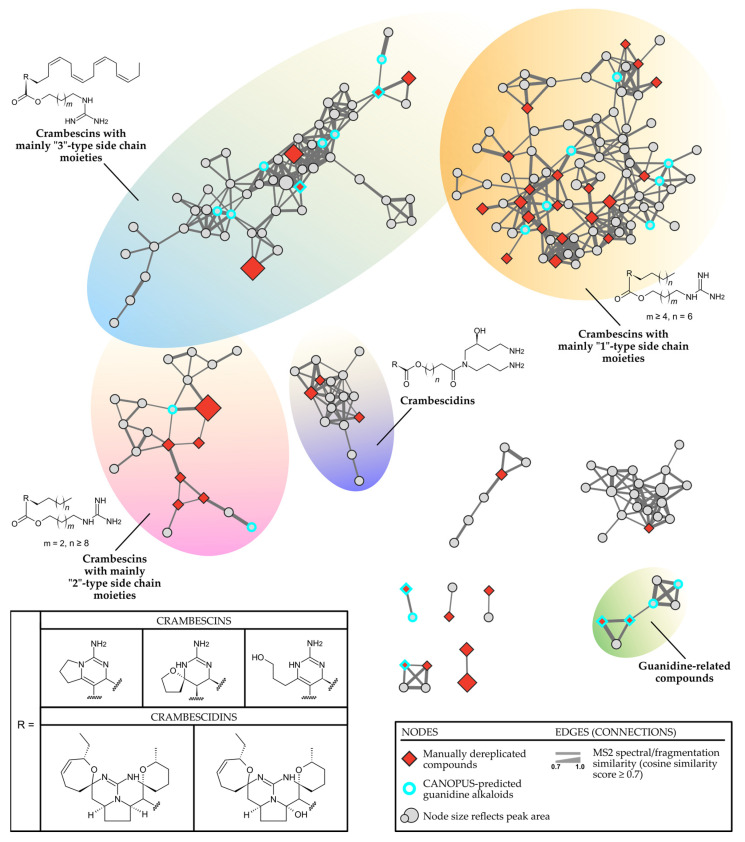
Feature-based molecular networking (FBMN) of the *Crambecrambe* extract visualized in Cytoscape. The legend can be referred to for an explanation of the colours and shapes of the nodes, along with details about the clusters. The MN was visually enhanced using the InkScape vector graphics editor [39].

The 53 annotated metabolites represent the dominant features as illustrated in Figure 2. When visualized via molecular networking, they form the basis for four distinct clusters of crambescin-related compounds (Figure 3). The GNPS2 workflow enabled the construction of a feature-based molecular network (FBMN) comprising 1024 nodes (consensus MS2 spectra) and 2629 edges. Subsequently, integrated SIRIUS results were mapped onto the MN, highlighting the compound classes present in the *C. crambe* extract, as predicted by the SIRIUS built-in tool CANOPUS. The visualized results indicated the presence of guanidine alkaloid compounds in the *C. crambe* extract, aligning with both previous findings [37] and the existing literature [11,38]. As shown in Figure 3, the overlay of manually dereplicated data onto the constructed FBMN revealed a compelling clustering pattern. Specifically, multiple guanidine-containing clusters were observed, collectively consisting of 223 nodes and connected with 537 edges.

Analysis of the molecular network suggests that the manually dereplicated A, B, and C crambescins form three primary clusters based on their side chain moieties (“1”, “2”, or “3”), rather than their core structures (monocyclic, bicyclic, or spiro-ring). Three major clusters emerged: the first, mainly composed of crambescins A1, B1, and C1; the second, primarily featuring A2, B2, and C2; and the third predominantly consisting of A3, B3, and C3 crambescins.

One of the clusters, containing 87 nodes connected by 227 edges, encompassed nodes annotated as CANOPUS-predicted guanidine alkaloids, as well as nodes associated primarily with crambescins with “1”-type side chain moieties (crambescins A1, B1, and C1). Another cluster, comprising 55 nodes connected by 149 edges, also included guanidine alkaloids, along with crambescins with “3”-type side chain moieties (crambescins A3, B3 and C3). A third characteristic guanidine-alkaloid-containing cluster (21 nodes and 29 edges) encompassed nodes corresponding to crambescins with “2”-type side chain moieties (crambescins A2, B2, and C2). Finally, two additional clusters were observed, containing features corresponding to crambescidins and other guanidine-like compounds.

The MN inspection confirmed that guanidine-containing clusters, as predicted by CANOPUS, include most nodes linked to the manually dereplicated compounds of interest. This finding paves the way for exploring adjacent “unknown” nodes. Additionally, the visual analysis successfully differentiated clusters of crambescins, crambescidins, and guanidine-related compounds.

**Table 2:** Compounds detected in *C. crambe* extract by ESI(+)-UHPLC-HRMS/MS.

#	*m*/*z*	Rt	Charge State, *z*	M_w_exp. ^a^	Proposed Formula	Δ(ppm) ^b^	Major MS/MS Fragments: *m*/*z* (Charge State, *z*)	Proposed Identification	Ref.
1	282.1806	8.16	1	281.1728	C_14_H_23_N_3_O_3_	−2.28	246.1587 (1); 264.1706 (1); 114.9612 (1); 60.0562 (1)	guanidine-related compound (C_14_H_23_N_3_O_3_)	
2	282.1806	9.34	1	281.1728	C_14_H_23_N_3_O_3_	−2.13	114.9611 (1); 60.0562 (1); 223.1323 (1)	guanidine-related compound (C_14_H_23_N_3_O_3_)	
3	264.1704	9.66	1	263.1626	C_14_H_21_N_3_O_2_	−0.99	246.1604 (1); 60.0562 (1)	guanidine-related compound (C_14_H_21_N_3_O_2_)	
*IS*	355.2012	9.80	1	354.1934	C_21_H_26_N_2_O_3_	−0.75	144.0807 (1); 212.1274 (1)	Yohimbine	
4	296.1964	10.44	1	295.1886	C_15_H_25_N_3_O_3_	−1.52	237.1476 (1); 205.1217 (1); 60.0562 (1); 159.1160 (1)	guanidine-related compound (C_15_H_25_N_3_O_3_)	
5	227.1803	10.46	2	452.3450	C_23_H_44_N_6_O_3_	−3.31	174.1600 (1); 148.6021 (2)	crambescin C 452 homologue (*m* = 5, *n* = 4)	
6	234.1884	10.77	2	466.3612	C_24_H_46_N_6_O_3_	−1.93	188.1756 (1); 155.6100 (2)	crambescin C 466 homologue (*m* = 6, *n* = 4)	
7							174.1600 (1); 148.6021 (2)	crambescin C 466 homologue (*m* = 5, *n* = 5)	
8	218.1757	10.87	2	434.3357	C_23_H_42_N_6_O_2_	−0.65	197.1646 (2); 174.1600 (1); 148.1095 (2); 220.1689 (1)	crambescin A 434 homologue (*m* = 5, *n* = 4)	
9	227.1803	10.88	2	452.3450	C_23_H_44_N_6_O_3_	−3.32	128.1431 (1); 174.1599 (1); 111.0442 (1); 284.1956 (1)	crambescin B 452 homologue (*m* = 5, *n* = 4)	
10	234.1884	10.98	2	466.3612	C_24_H_46_N_6_O_3_	−1.91	160.1441 (1); 141.5942 (2)	crambescin C1 466 (*m* = 4, *n* = 6)	[11]
11	241.1961	11.14	2	480.3765	C_25_H_48_N_6_O_3_	−2.42	188.1756 (1); 155.6096 (2)	crambescin C 480 homologue (*m* = 6, *n* = 5)	
12	225.1835	11.16	2	448.3514	C_24_H_44_N_6_O_2_	−0.45	204.1722 (2); 188.1756 (1); 155.1178 (2); 220.1689 (1)	crambescin A 448 homologue (*m* = 6, *n* = 4)	
13	292.8887	11.18	3	875.6426	C_44_H_87_N_6_O_11_	1.73	246.1587 (1); 139.0751 (1); 162.1598 (1); 381.3460 (1)	crambescidin 875	
14	234.1884	11.18	2	466.3612	C_24_H_46_N_6_O_3_	−1.93	128.1432 (1); 111.0443 (1); 188.1757 (1); 298.2129 (1)	crambescin B 466 homologue (*m* = 6, *n* = 4)	
15	279.2133	11.19	3	834.6166	C_45_H_82_N_6_O_8_	−1.01	246.1586 (1); 264.1707 (1); 70.0657 (1); 139.0750 (1) ^d^	crambescidin 834	
16	241.1961	11.24	2	480.3765	C_25_H_48_N_6_O_3_	−2.46	174.1600 (1); 148.6021 (2)	crambescin C1 480 (*m* = 5, *n* = 6)	[11]
17	218.1757	11.27	2	434.3357	C_23_H_42_N_6_O_2_	−0.58	127.0864 (2); 197.1646 (2); 132.1130 (1); 262.2150 (1)	crambescin A2 434 (*m* = 2, *n* = 7)	
18	249.1829	11.33	2	496.3502	C_28_H_44_N_6_O_2_	−2.79	114.1028 (1); 384.2650 (1); 132.1131 (1); 228.1724 (2)	crambescin A3 496 (*m* = 2) (cis)	
19	225.1834	11.36	2	448.3513	C_24_H_44_N_6_O_2_	−0.78	204.1722 (2); 141.1018 (2); 160.1441 (1); 248.2002 (1)	crambescin A1 448 (*m* = 4, *n* = 6)	
20	234.1884	11.43	2	466.3612	C_24_H_46_N_6_O_3_	−1.93	132.1131 (1); 127.5783 (2)	crambescin C2 466 (*m* = 2, *n* = 8)	
21							156.1748 (1); 111.0443 (1); 160.1441 (1)	crambescin B1 466 (*m* = 4, *n* = 6)	[11]
22	248.2038	11.49	2	494.3921	C_26_H_50_N_6_O_3_	−2.46	188.1756 (1); 155.6091 (2)	crambescin C1 494 (*m* = 6, *n* = 6)	[11]
23	238.1909	11.51	2	474.3662	C_26_H_46_N_6_O_2_	−2.02	188.1756 (1); 217.1806 (2); 155.1178 (2); 170.1650 (1)	didehydrocrambescin A1 474 (*m* = 6, *n* = 6)	
24	241.1960	11.55	2	480.3765	C_25_H_48_N_6_O_3_	−2.58	142.1585 (1); 111.0443 (1); 188.1756 (1); 298.2106 (1)	crambescin B 480 homologue (*m* = 6, *n* = 5)	
25	256.1910	11.55	2	510.3664	C_29_H_46_N_6_O_2_	−1.57	128.1178 (1); 384.2652 (1); 235.1809 (2); 146.1287 (1)	crambescin A3 510 (*m* = 3) (cis)	
26	249.1829	11.62	2	496.3502	C_28_H_44_N_6_O_2_	−2.73	132.1131 (1); 114.1028 (1); 127.0864 (2); 384.2638 (1)	crambescin A3 496 (*m* = 2) (trans)	
27	232.1909	11.62	2	462.3663	C_25_H_46_N_6_O_2_	−1.99	211.1798 (2); 174.1599 (1); 148.1093 (1); 248.2003 (1)	crambescin A1 462 (*m* = 5, *n* = 6)	[11]
28	241.1961	11.68	2	480.3765	C_25_H_48_N_6_O_3_	−2.46	156.1749 (1); 111.0443 (1); 174.1600 (1); 284.1957 (1)	crambescin B1 480 (*m* = 5, *n* = 6)	[11]
29	248.2039	11.68	2	494.3921	C_26_H_50_N_6_O_3_	−2.40	132.1131 (1); 127.0865 (2); 114.1026 (1)	crambescin C2 494 (*m* = 2, *n* = 10)	
30	225.1835	11.70	2	448.3513	C_24_H_44_N_6_O_2_	−0.64	127.0863 (2); 204.1721 (2); 132.1130 (1); 276.2321 (1)	crambescin A2 448 (*m* = 2, *n* = 8)	[11]
31	272.2038	11.71	2	542.3920	C_30_H_50_N_6_O_3_	−2.40	160.1441 (1); 142.1335 (1)	crambescin C3 542 (*m* = 4)	[12]
32	267.8778	11.73	3	800.6100	C_45_H_80_N_6_O_6_	−2.50	70.0657 (1); 349.2648 (2); 206.1536 (1); 392.3078 (2) ^d^	crambescidin 800 or isocrambescidin 800	[13,14]
33	263.1990	11.79	2	524.3824	C_30_H_48_N_6_O_2_	−0.76	142.1334 (1); 384.2649 (1); 160.1440 (1); 242.1875 (2)	crambescin A3 524 (*m* = 4) (cis)	[12]
34	256.1910	11.81	2	510.3664	C_29_H_46_N_6_O_2_	−1.65	146.1287 (1); 134.0943 (2); 128.1178 (1); 235.1805 (2)	crambescin A3 510 (*m* = 3) (trans)	
35	286.8851	11.83	3	857.6318	C_44_H_85_N_6_O_10_	1.41	264.1703 (1); 139.0749 (1); 246.1602 (1); 381.3456 (1)	crambescidin 857	
36	273.2094	11.84	3	816.6048	C_45_H_80_N_6_O_7_	−2.53	264.1707 (1); 246.1587 (1); 139.0751 (1); 70.0657 (1) ^d^	crambescidin 816	[14]
37	234.1884	11.88	2	466.3613	C_24_H_46_N_6_O_3_	−1.77	184.2054 (1); 132.1131 (1); 114.1028 (1); 242.1484 (1)	crambescin B2 466 (*m* = 2, *n* = 8)	
38	239.1989	11.90	2	476.3821	C_26_H_48_N_6_O_2_	−1.39	188.1756 (1); 156.174 (1); 111.0443 (1); 218.1873 (2)	crambescin A1 476 (*m* = 6, *n* = 6)	
39	281.5530	11.94	3	841.6355	C_44_H_85_N_6_O_9_	−0.20	263.1982 (1); 70.0657 (1); 116.1071 (1); 139.0751 (1)	crambescidin 841	
40	267.8778	11.94	3	800.6099	C_45_H_80_N_6_O_6_	−2.58	70.0657 (1); 349.2647 (2); 392.3078 (2); 206.1537 (1) ^d^	crambescidin 800 or isocrambescidin 800	[13,14]
41	248.2039	11.94	2	494.3921	C_26_H_50_N_6_O_3_	−2.34	156.1749 (1); 111.0444 (1); 188.1756 (1); 298.2130 (1)	crambescin B1 494 (*m* = 6, *n* = 6)	[11]
42	232.1910	11.98	2	462.3664	C_25_H_46_N_6_O_2_	−1.73	211.1799 (2); 134.0942 (2); 146.1287 (1); 276.2325 (1)	crambescin A 462 homologue (*m* = 3, *n* = 8)	
43	238.1908	11.98	2	474.3659	C_26_H_46_N_6_O_2_	−2.74	127.0864 (2); 132.1131 (1); 217.1807 (2); 114.1028 (1)	didehydrocrambescin A2 474 (*m* = 2, *n* = 10)	
44	270.2066	12.03	2	538.3977	C_31_H_50_N_6_O_2_	−1.36	497.3828 (1); 384.2652 (1); 522.3766 (1); 174.1602 (1) ^c^	crambescin A3 538 (*m* = 5) (cis)	[12]
45	263.1990	12.03	2	524.3825	C_30_H_48_N_6_O_2_	−0.63	160.1441 (1); 141.1018 (2); 242.1877 (2); 384.2650 (1)	crambescin A3 524 (*m* = 4) (trans)	[12]
46	232.1910	12.11	2	462.3664	C_25_H_46_N_6_O_2_	−1.82	127.0864 (2); 211.1799 (2); 132.113 (1); 290.2473 (1)	crambescin A2 462 (*m* = 2, *n* = 9)	[11]
47	416.3195	12.16	2	830.6233	C_46_H_82_N_6_O_7_	0.98	264.1707 (1); 246.1587 (1); 70.0657 (1); 139.0751 (1)	crambescidin 830	[14]
48	272.2037	12.18	2	542.3917	C_30_H_50_N_6_O_3_	−2.92	160.1441 (1); 111.0443 (1); 232.2046 (1); 274.2275 (1)	crambescin B3 542 (*m* = 4)	[12]
49	270.2066	12.27	2	538.3976	C_31_H_50_N_6_O_2_	−1.49	174.1597 (1); 148.1098 (2); 249.1955 (2); 156.1493 (1)	crambescin A3 538 ( *m* = 5) (trans)	[12]
50	404.2534	12.32	1	403.2463	C_22_H_33_O_4_N_3_	−1.74	360.2640 (1); 206.1536 (1); 342.2542 (1); 60.0562 (1)	crambescidin acid	[37]
51	239.1988	12.51	2	476.3820	C_26_H_48_N_6_O_2_	−1.65	127.0864 (2); 218.1873 (2); 132.113 (1); 304.2617 (1)	crambescin A2 476 (*m* = 2, *n* = 10)	[11]
52	254.2221	12.51	1	253.2148	C_14_H_27_N_3_O	−1.97	195.1740 (1); 97.0651 (1); 60.0562 (1); 111.0442 (1)	crambescin 253	[37]
*IS*	609.2800	12.59	1	608.2722	C_33_H_40_N_2_O_9_	−0.22	195.0642 (1); 174.0913; 397.2098 (1); 448.1952 (1)	reserpine	
53	282.2534	13.77	1	281.2460	C_16_H_31_N_3_O	−1.42	114.9612 (1); 223.2051 (1); 97.0651 (1); 60.0562 (1)	crambescin 281	[37]

^a^ Experimental molecular weight was calculated from the observed *m*/*z* as follows: $M_{w}calc.=\left(m/z\right)\times z-(z\times M_{H})$ where M_H_ = 1.0078 Da. ^b^ Mass error between the molecular weight was calculated from the experimental *m*/*z* and from the theoretical formula as follows: $\Delta \left(\mathrm{ppm}\right)=10^{6}\times \frac{\left(M_{w}\;\exp.-M_{w}\;\mathrm{theor}.\right)}{M_{w}\;\mathrm{theor}.}$. For determination of the theoretical molecular weight, the following monoisotopic mass values were considered for each element of H, C, *n*, and O: M_H_ = 1.0078 Da, M_C_ = 12 Da, M_N_ = 14.0031 Da, and M_O_ = 15.9949 Da. [40].^c^ MS/MS data-dependent scans were not triggered on the [M+2H]^2^ ion. Data from the MS/MS fragmentation of the [M+H]^+^ion are reported. ^d^ Data from the MS/MS fragmentation of the [M+2H]^2+^ ion are reported.

### 2.1. Crambescin A

**Figure 4:**
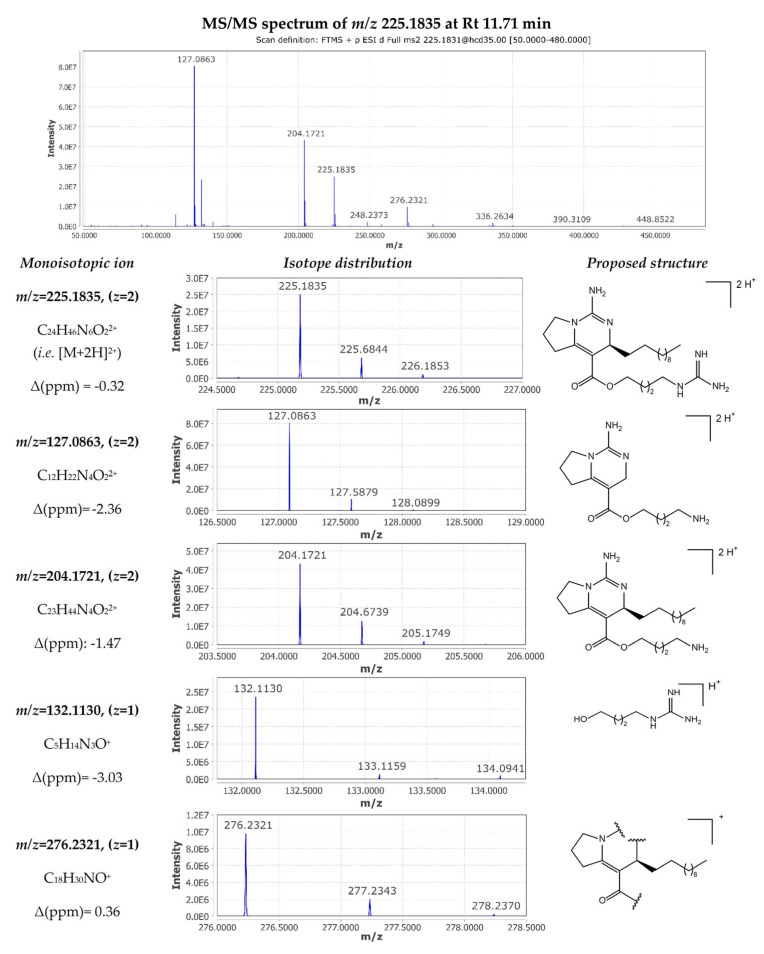
MS/MS fragmentation of crambescin A2 448 (compound **30**).

The most abundant metabolite of the extract (compound **30**) was detected at 11.71 min with *m*/*z* 225.1835 corresponding to [M+2H]^2+^ consistent with a molecular formula of C_24_H_44_N_6_O_2_. In the MN analysis, the corresponding feature node is a part of the crambescin “2”-type sidechain moiety cluster, connected with another A2 crambescin (compound **51**) and an annotated node congruent with CANOPUS-derived predictions for guanidine compounds. The single-charged ion [M+H]^+^ was also observed at *m*/*z* 449.3600 (Δ 1.34 ppm) with an intensity 50 times lower than that of the double-charged ion. The MS/MS spectrum of the [M+2H]^2+^ ion displayed characteristic fragments which provided insights on both alkyl and guanidinoalkyl side chains (Figure 4). More specifically, the loss of methylenediamine (−42 Da, CH_2_N_2_) yielded in the *m*/*z* 204.1721 fragment ion ([C_23_H_44_N_4_O_2_]^2+^, Δ −1.47 ppm), and further dissociation of the aliphatic side chain (−154 Da, C_11_H_22_) resulted in *m*/*z* 127.0863 ([C_12_H_22_N_4_O_2_]^2+^, Δ −2.36 ppm). Therefore, double-charged fragments can serve to determine the length of the aliphatic side chain at C-13. On the other hand, single-charged fragment *m*/*z* 132.1130 ([C_5_H_14_N_3_O]^+^, Δ −3.03 ppm) provided a direct characterization of the guanidinoalkyl side chain. Finally, the fragment ion *m*/*z* 276.2321 ([C_18_H_30_NO]^+^, Δ 0.36 ppm) could be related to the cleavage of both the guanidine cyclic core and guanidine ester. Consequently, compound **30** was identified as crambescin A2 448.

Interestingly, this fragmentation pattern led to the characterization of new homologues from the crambescin A family, which displayed different ratios regarding the number of methylene units composing the alkyl side chains. MS/MS spectra of compound **8** were consistent with a crambescin A 434 homologue with *m* = 5 and *n* = 4. Double-charged fragments with *m*/*z* 197.1646 and 148.1095 corresponded to [C_22_H_42_N_4_O_2_]^2+^ and [C_15_H_28_N_4_O_2_]^2+^, respectively (Δ, −0.76 and −2.70 ppm). As mentioned previously, neutral loss between these two fragments corresponded to the dissociation of the C-13 alkyl side chain, i.e., C_7_H_14_, while the guanidinoalkyl chain was characterized by the fragment *m*/*z* 174.1600 ([C_8_H_20_N_3_O]^+^, Δ −1.15 ppm).

Analogous to this fragmentation pattern, 10 compounds of the crambescin A group were annotated. Compounds **17** and **19** were identified as crambescin A2 434 and crambescin A1 448. Crambescin A1 462 and crambescin A2 462 were assigned to compounds **27** and **46** while compounds **38** and **51**, respectively, corresponded to crambescin A1 476 and crambescin A2 476. The oxidized forms of **38** and **51**, which were consistent with a formula of C_24_H_46_N_6_O_2_, were detected at 11.51 min (didehydrocrambescin A1 474, compound **23**) and at 11.98 min (didehydrocrambescin A2 474, compound **43**).

Additionally, another two new crambescin A homologues were characterized, compounds **12** and **42**, which were identified as crambescin A 448 (*m* = 6, *n* = 4) and crambescin A 462 (*m* = 3, *n* = 8), respectively.

**Figure 5:**
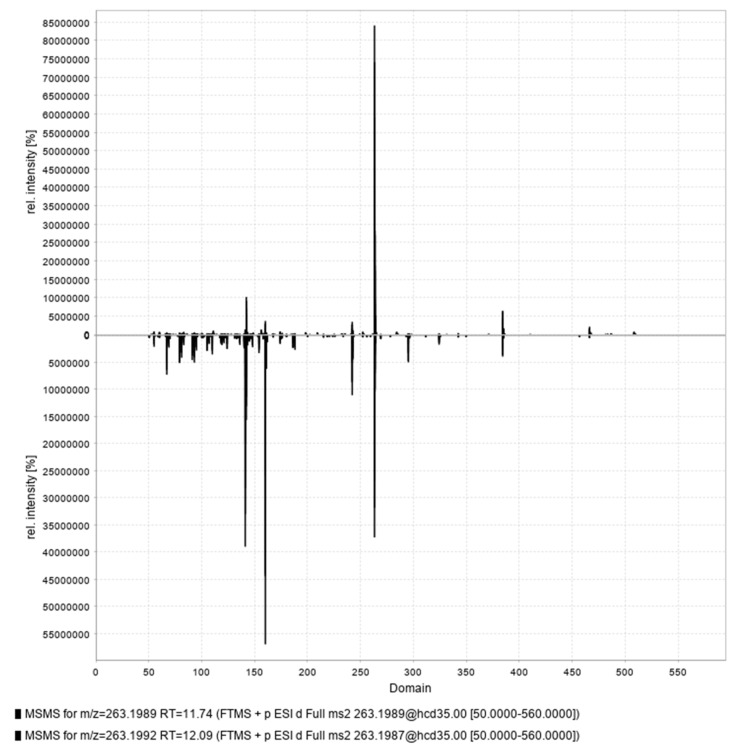
Comparison of MS/MS spectra of cis and trans isomers of crambescin A3 524 (compound **33** and **45**, respectively).

A series of compounds presenting empirical molecular formulae of the type C_n_H_2n-12_N_6_O_2_ were found to belong to the crambescin A3 sub-family, and were first introduced by Genta-Jouve et al. [41]. These metabolites exhibited Rings and Double Bond Equivalents (RDBEs) of 10 which indicates an extra four degrees of unsaturation compared to other crambescin A types (C_n_H_2n-4_N_6_O_2_, RDBE 6). Following a similar fragmentation pathway, a neutral loss of 202 Da (-C_15_H_22_) between double-charged fragment ions enabled us to confirm that these additional unsaturated bonds were located on the upper aliphatic side chain. Consequently, compounds **18** and **26** were determined to be crambescin A3 496 isomers, compounds **25** and **34** were found to be crambescin A3 510 isomers, compounds **44** and **49** were identified as crambescin A3 538 isomers, and compounds **33** and **35** were assigned to crambescin A3 524 isomers. To the authors’ knowledge, it is the first time that crambescin A3 isomerism has been reported. The same characteristic fragment ions were observed for each pair of isomers, differing only by their intensity as shown in Figure 5. The annotation of *cis* and *trans* isomers was based on their expected relative polarity.

### 2.2. Crambescins B and C

In addition to crambescin A, the investigation of the *C. crambe* metabolic profile revealed the presence of 16 compounds belonging to crambescin B and C families, which exhibited a different fragmentation pattern. These analogues display empirical molecular formulae of the type C_n_H_2n_-2N_6_O_3_, and their discrimination was based on the intensity of specific fragment ions. Due to the presence of two conjugated double bonds in the guanidine ring, members of the crambescin C family may undergo a retro-Diels–Alder reaction less easily compared to crambescin B, which possesses only one intracyclic bond [42].

**Figure 6:**
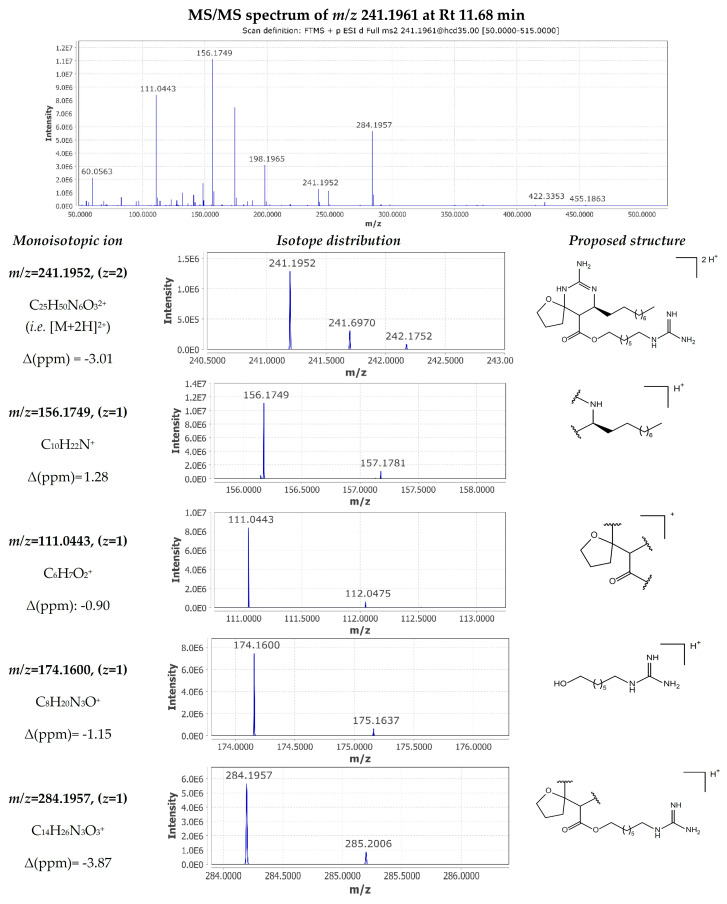
MS/MS fragmentation of crambescin B1 480 (compound **28**).

A pair of isomers consistent with a molecular formula of C_25_H_48_N_6_O_3_ were detected at 11.24 min and 11.68 min (compounds **16** and **28**). Following the analysis of the MS/MS spectrum presented in Figure 6, the double-charged ion [M+2H]^2+^ with *m*/*z* 241.1961 observed at 11.68 min was identified as the known metabolite crambescin B1 480 (compound **28**). The guanidine cyclic core underwent a pericyclic reaction via a retro-Diels–Alder mechanism yielding two fragment ions *m*/*z* 198.1965 ([C_11_H_24_N_3_]^+^, Δ 0.00 ppm) and *m*/*z* 284.1957 ([C_14_H_26_N_3_O_3_]^+^, Δ −3.87 ppm). Consequently, the loss of methylenediamine from the first fragment resulted in *m*/*z* 156.1749 ([C_10_H_22_N]^+^, Δ 1.28 ppm), while dissociation of the guanidine ester from the second fragment produced the *m*/*z* 111.0443 ([C_6_H_7_O_2_]^+^, Δ −0.90 ppm) and *m*/*z* 174.1600 ([C_8_H_20_N_3_O]^+^ Δ −1.15 ppm) assigned to the spiroaminal ring and the lower guanidoalkyl chain, respectively. The constructed FBMN clustered crambescin B1 480 with crambescins B1 494, C1 466, and C1 480, creating a larger constellation. The manual dereplication method indicated that the majority of these compounds feature “1”-type moieties or side chains, regardless of the central cyclic guanidine core. Additionally, all these nodes are positioned within the previously mentioned subnetwork that includes CANOPUS-predicted guanidine compounds.

**Figure 7:**
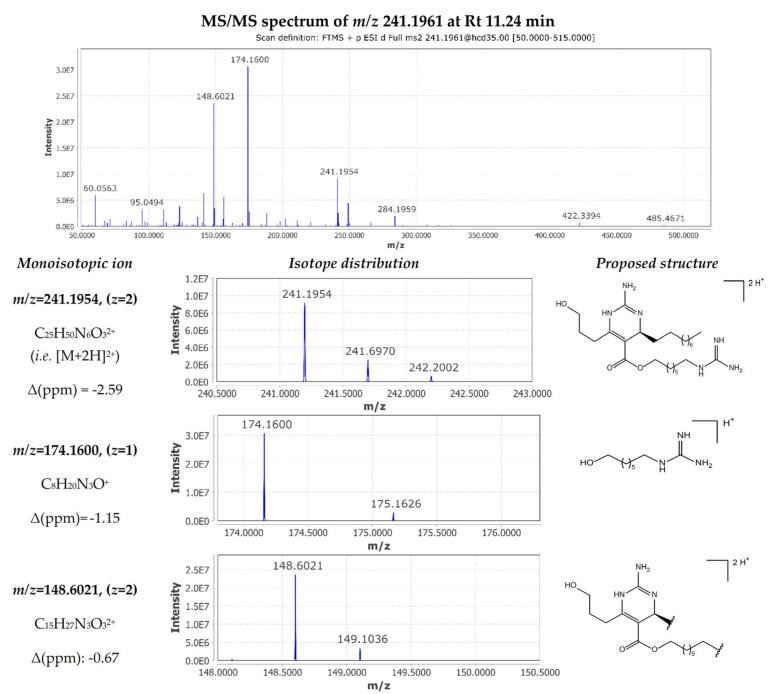
MS/MS fragmentation of crambescin C1 480 (compound **16**).

The fragmentation pattern observed for the metabolite corresponded to the double-charged ion [M+2H]^2+^ with *m*/*z* 241.1961 at 11.24 min and was very similar to that of crambescin B1 480. MS/MS spectrum inspection revealed the presence of the same fragments at significantly lower intensities. However, the difference lies in the presence of an intense double-charged fragment ion *m*/*z* 148.6021 ([C_15_H_27_N_3_O_3_]^2+^ Δ −0.67 ppm), implying the losses of the upper aliphatic chain and the guanidine moiety of the lower chain. Moreover, the other major fragment with *m*/*z* 174.1600 ([C_8_H_20_N_3_O]^+^ Δ −1.15 ppm) allowed the determination of the length of the lower aliphatic chain (Figure 7). Based on these observations, compound **16** was identified as crambescin C1 480.

Another pair of isomers eluting at 11.14 min (compound **11**) and 11.55 min (compound **24**) presented the same molecular formula with crambescins B1 and C1 480. MS/MS spectra of these metabolites displayed the same fragmentation motives, varying only in the length of the side chains. As a result, two new analogues were identified belonging to the crambescin B and C groups, which bore the same number of methylene units in the lower and upper aliphatic chain (*m* = 6, *n* = 5).

Based on the characteristic fragmentation scheme of crambescins B and C, other analogues were annotated accordingly. Compounds **22**, **29**, and **41** were identified as crambescins C1, C2, and B1 494, respectively, compounds **10** and **21** were assigned to crambescins C1 and B1 466, and compounds **20** and **37** corresponded to crambescins C2 and B2 466. Moreover, five new analogues were reported for the first time including two crambescin C 466 homologues (*m* = 6, *n* = 4; compound **6** and *m* = 5, *n* = 5; compound **7**), one crambescin B 466 homologue with *m* = 6, *n* = 4 (compound **14**), and one crambescin C 452 homologue (*m* = 5, *n* = 4; compound **5**), as well as one crambescin B 452 homologue with *m* = 5, *n* = 4 (compound **9**). Mass spectra and detailed fragmentation patterns for these new analogues are presented in the Supplementary Information (Supplementary Material, Figures S1–S10, Tables S1–S10).

Finally, two compounds presenting a molecular formula of C_30_H_50_N_6_O_3_ indicating 9 degrees of unsaturation were found to be the known crambescins C3 and B3 542 (compounds **31** and **48**, respectively) [41] along with two minor metabolites, crambescins B 253 and 281 (compounds **52** and **53**) [37].

### 2.3. Crambescidins and Other Guanidine Alkaloids

A triple-charged ion *m*/*z* 273.2094 eluting at 11.84 min was accounted for among the major metabolites detected in the extract. This feature was consistent with a molecular formula of C_45_H_80_N_6_O_7_ and was assigned to be crambescidin 816 (compound **37**), a member of the crambescidin family that was introduced by Jares-Erijman et al. [14]. Other crambescidin metabolites present at lower concentrations were also identified including isomeric forms of crambescidin 800 (compounds **32** and **40**) and crambescidin 830 (compound **47**). Moreover, four new crambescidin analogues were detected, compound **13** (crambescidin 875, molecular formula C_44_H_87_N_6_O_11_), compound **15** (crambescidin 834, molecular formula C_45_H_82_N_6_O_8_), compound **35** (crambescidin 857, molecular formula C_44_H_85_N_6_O_10_), and compound **39** (crambescidin 841, molecular formula C_44_H_85_N_6_O_9_).

At the MS level, the base peak ion of all crambescidin analogues typically corresponded to the [M+3H]^3+^ form, with the [M+2H]^2+^ ion being present at lower intensities. At the MS/MS level, characteristic fragment ions were observed for all the compounds mentioned here above. More specifically, the fragment ion *m*/*z* 264.17 ([C_14_H_22_N_3_O_2_]^+^, Δ −1.51 to 0.00 ppm) corresponded to the guanidine core after the loss of the ethyl oxepine moiety and further dehydration yielded *m*/*z* 246.16. Another common fragment ion, *m*/*z* 70.0657 ([C_4_H_8_N]^+^, Δ 2.85 ppm), was produced by the fragmentation of the hydroxyspermidine unit and subsequent cleavage of the hydroxyl group. However, the structural elucidation of these new crambescidin analogues was not feasible due to the high complexity of the MS/MS spectra and the insufficient ion abundance, which limited the reliability of fragmentation pattern analysis.

Additionally, four guanidine-related compounds of lower molecular weights were observed as single-charged ions potentially belonging to the crambescidin family (compounds **1**–**4**) along with the known crambescidin acid (compound **50**).

In the context of molecular networking, the manually dereplicated crambescidins, as well as guanidine-related compounds, form their own distinct clusters.

## 3. Materials and Methods

### 3.1. Chemicals

Analytical grade dichloromethane and methanol that were used for sample extraction were purchased from Sigma-Aldrich (Steinheim, Germany). For sample reconstitution of the crude extract and further UHPLC-HRMS analysis, LC-MS grade solvents were used. More specifically, acetonitrile (LC-MS grade) was purchased from Fluka/Riedel-de Haën (Seelze, Germany), methanol (LC-MS grade) was acquired from Sigma-Aldrich (Steinheim, Germany), and formic acid (LC-MS grade) was obtained from Thermo Fisher Scientific (Geel, Belgium). High-purity water was provided by a Millipore Milli-Q Plus water purification system (Millipore, Milford, MA, USA). Reserpine and yohimbine were purchased from Sigma-Aldrich (Steinheim, Germany).

### 3.2. Sample Preparation

A sample (wet weight 277g) from the *C. crambe* species (class: *Demospongiae*; order: *Poecilosclerida*) [43] was collected from the Mediterranean Sea, La Herradura, Spain, 36°43′23.149″ N 3°43′35.91″ W. It was then lyophilized and further extracted using the Accelerated Solvent Extraction (ASE) method with a mixture of dichloromethane and methanol (50:50, *v*/*v*). The crude extract was obtained after evaporation of the solvents under reduced pressure and was reconstituted in MeOH in order to obtain a concentration of 1 mg.mL^−1^. The reconstituted sample was vortexed thoroughly for 10 min and further subjected to centrifugation at 12,000 rpm for 10 min at 4 °C. The supernatant was diluted and spiked with a mixture of reserpine and yohimbine, used as internal standards. The final concentration was 0.1 μg/mL crude *C. crambe* extract and 270 μg/mL for each internal standard.

### 3.3. UHPLC-HRMS/MS Analysis

Liquid chromatography analysis was performed on a Dionex Ultimate 3000 UHPLC system and detection was carried out on a Q-Exactive Orbitrap mass spectrometer equipped with an HESI source (Thermo Scientific, Bremen, Germany). More specifically, separation was achieved on a Hypersil Gold UPLC C18 (2.1 × 100 mm, 1.9 μm) (Thermo Scientific) column heated at 40 °C. Solvent A was Milli-Q water with 0.1% (*v*/*v*) formic acid and solvent B was acetonitrile. Gradient elution started with 95% A, decreasing to 5% A in 24 min. These conditions were kept for 4 min before returning to the initial conditions for a 2 min re-equilibration. Auto-sampler tray temperature was maintained at 8 °C and the injection volume was 10 μL. HRMS data were acquired in both negative and positive modes, in the full scan *m*/*z* range of 110–1000, with a resolution of 70,000 FWHM in profile mode. Data-dependent acquisition was simultaneously performed using HCD fragmentation with a normalized collision energy of 35% and a mass resolution of 35,000. Capillary temperature was set at 320 °C in both polarities, whereas source voltage was 3.6 kV in ESI+ and 3.2 kV in ESI-. Probe Heater Temperature was 200 °C and the S-Lens RF Level was set at 55. Finally, nitrogen was used as the sheath gas (40 arbitrary units) and auxiliary gas (8 arbitrary units).

### 3.4. Molecular Networking/Computational Chemical Dereplication

UHPLC-HRMS/MS chromatograms were acquired using Xcalibur version 2.1 (Thermo Fisher Scientific).

For the MN-based dereplication, the obtained Thermo Fisher *.raw data were first converted to the *.mzML format with the use of MSConvert, a tool from the ProteoWizard suite [44,45], in order to be processed in the MZmine2 environment [30]. During the conversion, the data are centroided (transforming profile data into a format that represents peak intensities at discrete *m*/*z* values) by applying a peak-picking filter. Data processing involved the following steps and parametrizations: (a) Mass detection was based on a defined threshold of 100 for MS1 and 10 for MS2. (b) Chromatogram building was achieved by implementing the GridMass–2D module [46]. More specifically, the algorithm was set to generate a grid of equally spaced probes of 0.05 min and 0.05 Da in the entire time range (1st dimension) and the *m*/*z* range (2nd dimension) of the chromatogram. (c) Chromatogram deconvolution was achieved through a local minimum search module, with a chromatographic threshold of 1%, a minimum RT of 0.05, a minimum relative height of 1%, a minimum absolute height of 200, and a minimum ratio of peak to edge of 1. (d) Lastly, we applied feature filtering to only keep the ones with MS2-containing scans. Following MZmine processing, data were forwarded to the GNPS2 online platform for molecular network construction and feature prediction through library matching [34]. FBMN workflow parameters in the GNPS2 environment included the following: general parameters—precursor ion tolerance 0.02 Da; fragment ion tolerance 0.02; networking parameters—minimum cosine score 0.7; minimum matched peaks 6; library search parameters—minimum cosine score 0.7; minimum matched peaks 6; search for analogues enabled. Furthermore, the data were imported into the SIRIUS [31] environment to predict the identity and chemical classes of the compounds based on the implemented CSI: FingerID [32] and the Support Vector Machine algorithms of CANOPUS and ClassyFire [33,47]. The NPC ClassyFire results were visualized in sunburst plots through *Plotly* Python [48] and were also integrated into the GNPS2-constructed FBMN [27,34] by utilizing the robust Cytoscape environment [35]. The Cytoscape platform was utilized for the visualization of FBMN with a significant advantage being its ability to display any tabular data as attributes of the network, applicable to both nodes and edges. For the MN layout, the *yfiles* [49] organic style was chosen since it reveals inherent symmetric and clustered structures within undirected graphs while ensuring a balanced distribution of nodes and minimizing edge crossings. The acquired data and workflows are available under the Findability, Accessibility, Interoperability, and Reuse (FAIR) principles. Specifically, MS/MS data are stored in the MassIVE repository (massive.ucsd.edu) with the identifier MSV000096117. The MN output and its parametrization can be found on the GNPS repository (https://gnps2.org/status?task=2e9e2fe9d05743118d93cbb64f30acb0, accessed on 18 November 2024).

## 4. Conclusions

An in-depth qualitative investigation of the Mediterranean sponge *Crambecrambe* was performed using UHPLC-HRMS/MS. The study of the CH_2_Cl_2_-MeOH extract led to the annotation of 53 compounds belonging to the crambescin and crambescidin families. A computational mass spectrometry approach, based on molecular networking studies and Natural Product Class predictions (NPC), enabled the further study of the crambescin guanidine alkaloid family and the prioritization of compounds for future isolation. A detailed fragmentation pattern is proposed for the characterization of crambescin compounds, which enabled the identification of 22 analogues reported for the first time. Interestingly, MS/MS data analysis highlighted the presence of four new homologous series of crambescins, regarding the ratio of methylene units that compose the upper (*n* + 2) and lower (*m* + 2) alkyl side chains. These new homologues included crambescins B/C 452 (compounds **9**/**5**) and crambescin A 434 (compound **8**) with *m* = 5, *n* = 4; crambescin C 466 (compound **7**) with *m* = 5, *n* = 5; crambescins B/C 466 (compounds **14**/**6**) and crambescin A 448 (compound **12**) with *m* = 6, *n* = 4; and crambescins B/C 480 (compounds **24**/**11**) with *m* = 6, *n* = 5. Moreover, the presence of eight crambescidin compounds is reported, among which four of them constitute new analogues. Due to its sensitivity, the high mass accuracy at both MS and MS/MS levels, and the extensive structural information provided through HCD fragmentation, the implemented UHPLC-HRMS/MS methodology demonstrated its fitness for purpose regarding the characterization of both major and minor guanidine alkaloids from *C. crambe* sponge.

## Figures and Tables

**Table 1:** The proposed structures of 22 new crambescin analogues.

Proposed Structure	Proposed Identification/Compound No.
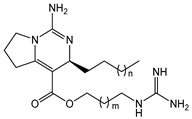	crambescin A 434 homologue (*m* = 5, *n* = 4)/compound **8**
	crambescin A 448 homologue (*m* = 6, *n* = 4)/compound **12**
	crambescin A 462 homologue (*m* = 3, *n* = 8)/compound **42**
	crambescin A1 448 (*m* = 4, *n* = 6)/compound **19**
	crambescin A1 476 (*m* = 6, *n* = 6)/compound **38**
	crambescin A2 434 (*m* = 2, *n* = 7)/compound **17**
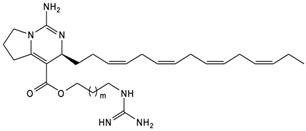	crambescin A3 496 (*m* = 2) (*cis*)/compound **18**
	crambescin A3 510 (*m* = 3) (*cis*)/compound **25**
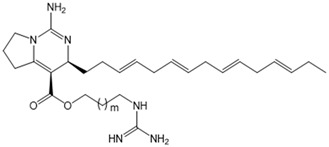	crambescin A3 496 (*m* = 2) (*trans*)/compound **26**
	crambescin A3 510 (*m* = 3) (*trans*)/compound **34**
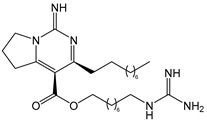	didehydrocrambescin A1 474 (*m* = 6, *n* = 6)/compound **23**
	didehydrocrambescin A2 474 (*m* = 2, *n* = 10)/compound **43**
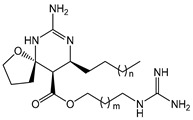	crambescin B 452 homologue (*m* = 5, *n* = 4)/compound **9**
	crambescin B 466 homologue (*m* = 6, *n* = 4)/compound **14**
	crambescin B 480 homologue (*m* = 6, *n* = 5)/compound **24**
	crambescin B2 466 (*m* = 2, *n* = 8)/compound **37**
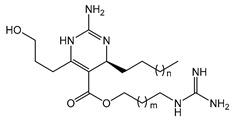	crambescin C 452 homologue (*m* = 5, *n* = 4)/compound **5**
	crambescin C 466 homologue (*m* = 5, *n* = 5)/compound **7**
	crambescin C 466 homologue (*m* = 6, *n* = 4)/compound **6**
	crambescin C 480 homologue (*m* = 6, *n* = 5)/compound **11**
	crambescin C2 466 (*m* = 2, *n* = 8)/compound **20**
	crambescin C2 494 (*m* = 2, *n* = 10)/compound **29**

## Data Availability

The acquired data and workflows are stored as mentioned above in the MassIVE repository (massive.ucsd.edu) with the identifier MSV000096117, whereas the MN output can be found on the GNPS repository (https://gnps2.org/status?task=2e9e2fe9d05743118d93cbb64f30acb0).

## Supplementary Materials

The following supporting information can be downloaded at https://www.mdpi.com/article/10.3390/md22110522/s1: Figure S1. MS spectrum of crambescin B 452 homologue (*m* = 5, *n* = 4), compound **9**; Table S1. Proposed structure of compound **9**; Figure S2. MS/MS spectrum of compound **9**; Table S2. Fragmentation of compound **9**; Figure S3. MS spectrum crambescin B 466 homologue (*m* = 6, *n* = 4), compound **14**; Table S3. Proposed structure of compound **14**; Figure S4. MS/MS spectrum of compound **14**; Table S4. Fragmentation of compound **14**; Figure S5. MS spectrum of crambescin C 452 homologue (*m* = 5, *n* = 4), compound **5**; Table S5. Proposed structure of compound **5**; Figure S6. MS/MS spectrum of compound **5**; Table S6. Fragmentation of compound **5**; Figure S7. MS spectrum of crambescin C 466 homologue (*m* = 6, *n* = 4), compound **6**; Table S7. Proposed structure of compound **6**; Figure S8. MS/MS spectrum of compound **6**; Table S8. Fragmentation of compound **6**; Figure S9. MS spectrum of crambescin C 466 homologue (*m* = 5, *n* = 5), compound **7**; Table S9. Proposed structure of compound **7**; Figure S10. MS/MS spectrum of compound **7**; Table S10. Fragmentation of compound **7**; Table S11. Structures of 53 annotated crambescin-, crambescidin-, and other guanidine-related compounds.
