# Supplementary material for: Chemical Investigation of the Mediterranean Sponge Crambe crambe by UHPLC-HRMS/MS via Manual and Computational Dereplication Approaches
Source: Mar Drugs. 2024 Nov 20;22(11):522. doi: 10.3390/md22110522 (PMC11595807; doi:10.3390/md22110522)
Supplement: Supplementary file 1 [file marinedrugs-22-00522-s001.zip › Supplementary Material Crambe crambe_final.pdf]

Supplementary Material

Chemical Investigation of the Mediterranean Sponge *Crambe crambe* by UHPLC-HRMS/MS via Manual and Computational Dereplication Approaches

Table of Contents

Figure S1. MS spectrum of Crambescin B 452 homologue (m=5, n=4), Compound 9 2
Table S1. Proposed structure of compound 9 2
Figure S2. MS/MS spectrum of compound 9 3
Table S2. Fragmentation of compound 9 3
Figure S3. MS spectrum Crambescin B 466 homologue (m=6, n=4), Compound 14 4
Table S3. Proposed structure of compound 14 4
Figure S4. MS/MS spectrum of compound 14 5
Table S4. Fragmentation of compound 14 5
Figure S5. MS spectrum of Crambescin C 452 homologue (m=5, n=4), Compound 5 6
Table S5. Proposed structure of compound 5 6
Figure S6. MS/MS spectrum of compound 5 7
Table S6. Fragmentation of compound 5 7
Figure S7. MS spectrum of Crambescin C 466 homologue (m=6, n=4), Compound 6 8
Table S7. Proposed structure of compound 6 8
Figure S8. MS/MS spectrum of compound 6 9
Table S8. Fragmentation of compound 6 9
Figure S9. MS spectrum of Crambescin C 466 homologue (m=5, n=5), Compound 7 10
Table S9. Proposed structure of compound 7 10
Figure S10. MS/MS spectrum of compound 7 11
Table S10. Fragmentation of compound 7 12
Table S11. Structures of 53 annotated crambescin-, crabescidin- and other guanidine-related compounds 13

Figure S1. MS spectrum of Crambescin B 452 homologue (m=5, n=4), Compound **9**

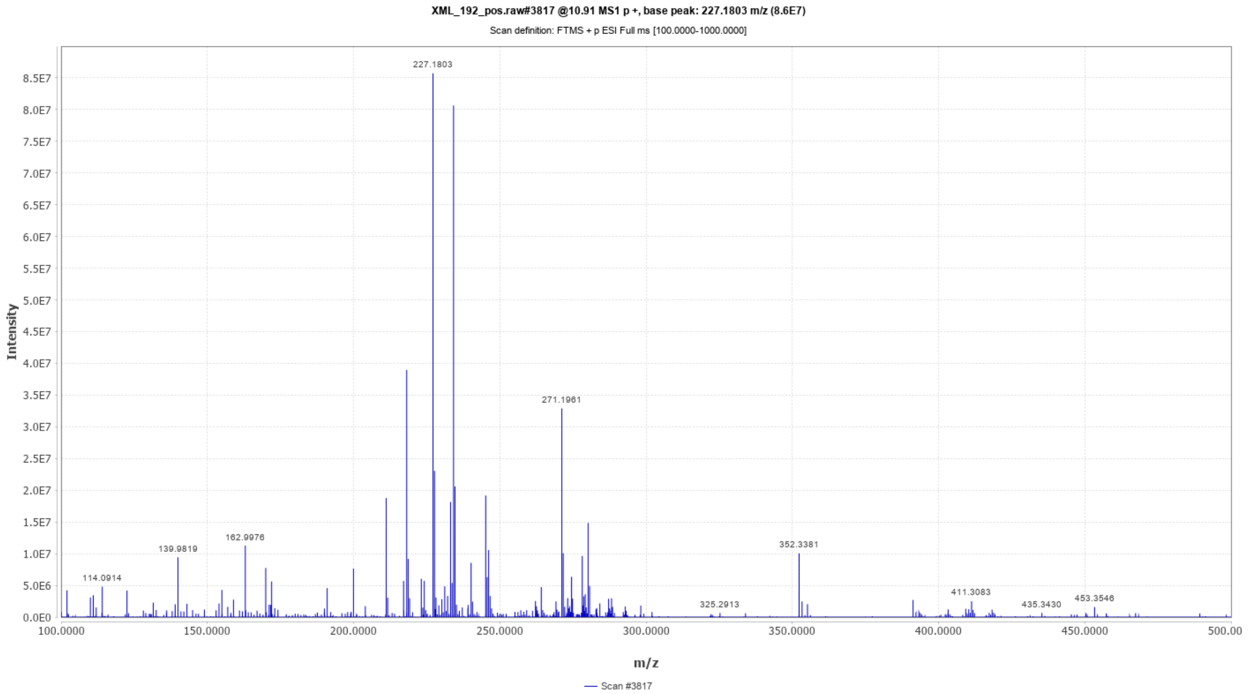

Table S1. Proposed structure of compound **9**

m/z	Rt	Charge state, *z*	M_w exp.*	Proposed Formula*	Δ(ppm) *	Proposed Structure [M+2H]$^{2+}$
227.1803	10.88	2	452.3450	$C_{23}H_{44}N_6O_3$	-3.32	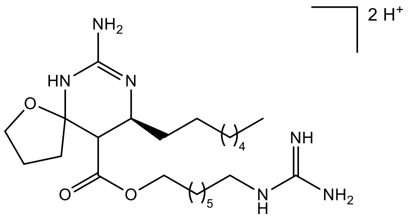

* *corresponding to the non-charged species, calculation of the experimental M_w and mass error are detailed in the note to* ***Table 2****.*

Figure S2. MS/MS spectrum of compound **9**

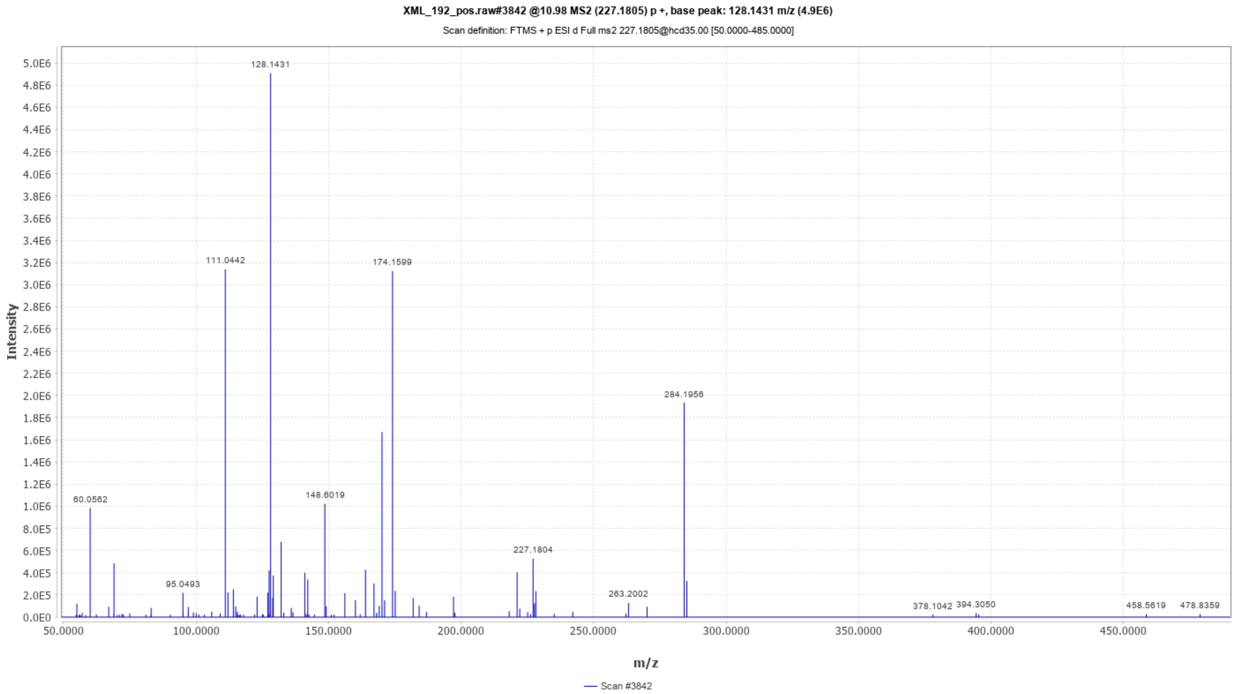

Table S2. Fragmentation of compound **9**

MS/MS fragment m/z	Charge state, *z*	Proposed Formula	Δ(ppm)	Proposed Structure
128.1431	1	$C_8H_{18}N^+$	-3.12	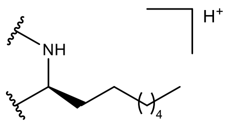
174.1599	1	$C_8H_{20}N_3O^+$	-1.72	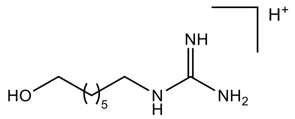
111.0442	1	$C_6H_7O_2^+$	-1.80	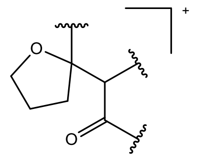
284.1956	1	$C_{14}H_{26}N_3O_3^+$	-4.22	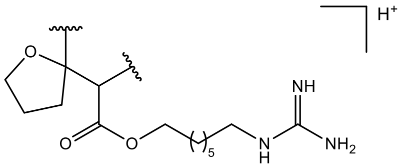
170.1649	1	$C_9H_{20}N_3^+$	-2.35	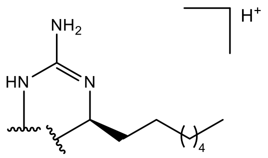

Figure S3. MS spectrum Crambescin B 466 homologue (m=6, n=4), Compound **14**

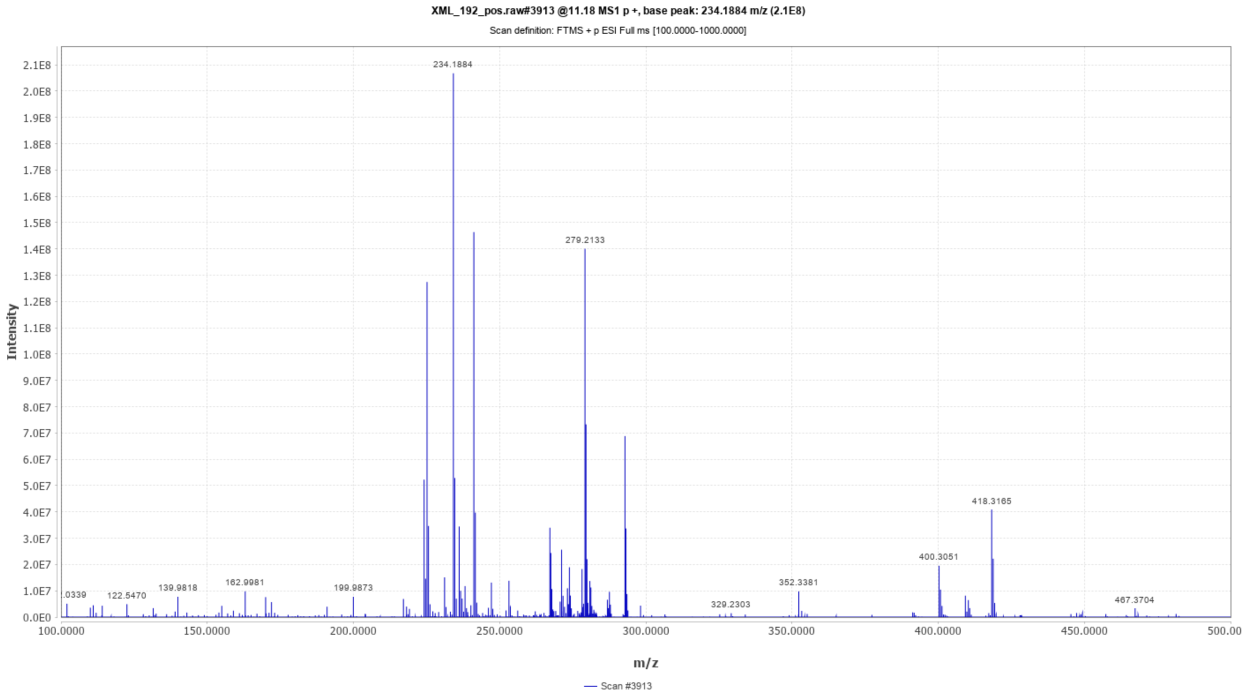

Table S3. Proposed structure of compound **14**

m/z	Rt	Charge state, *z*	Mw exp.*	Proposed Formula*	Δ(ppm) *	Proposed Structure [M+2H]$^{2+}$
234.1884	11.18	2	466.3612	$C_{24}H_{46}N_6O_3$	-1.93	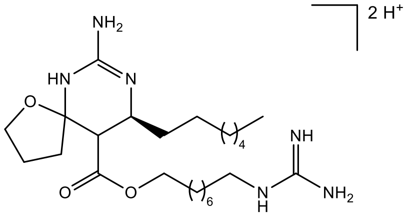

* *corresponding to the non-charged species, calculation of the experimental* M_w *and mass error are detailed in the note to* ***Table 2****.*

Figure S4. MS/MS spectrum of compound **14**

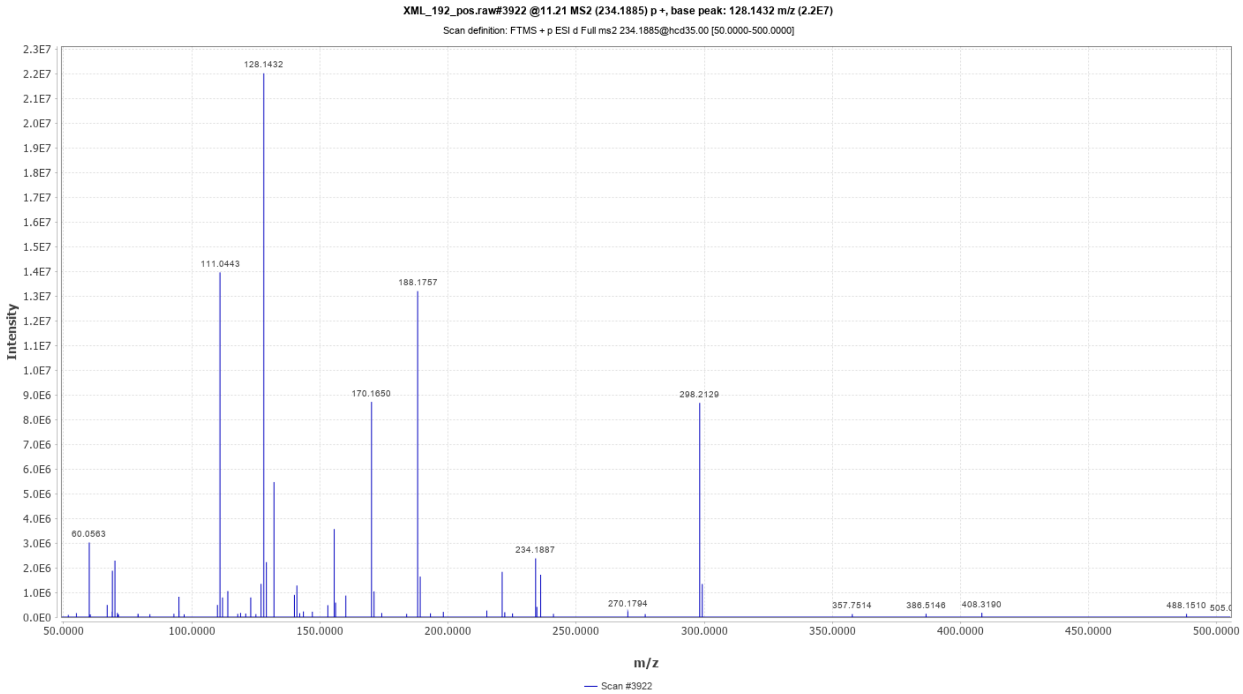

Table S4. Fragmentation of compound **14**

MS/MS fragment m/z	**Charge state, *z***	**Proposed Formula**	**Δ(ppm)**	**Proposed Structure**
128.1432	1	$C_8H_{18}N^+$	-2.34	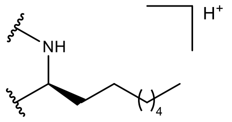
111.0443	1	$C_6H_7O_2^+$	-0.90	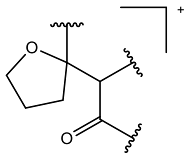
188.1757	1	$C_9H_{22}N_3O^+$	-0.53	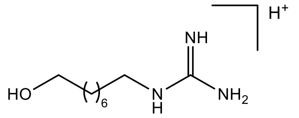
298.2129	1	$C_{15}H_{28}N_3O_3^+$	-1.68	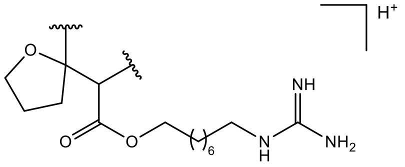
170.1650	1	$C_9H_{20}N_3^+$	-1.76	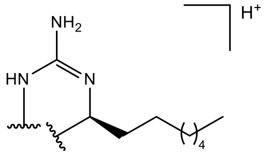

Figure S5. MS spectrum of Crambescin C 452 homologue (m=5, n=4), Compound **5**

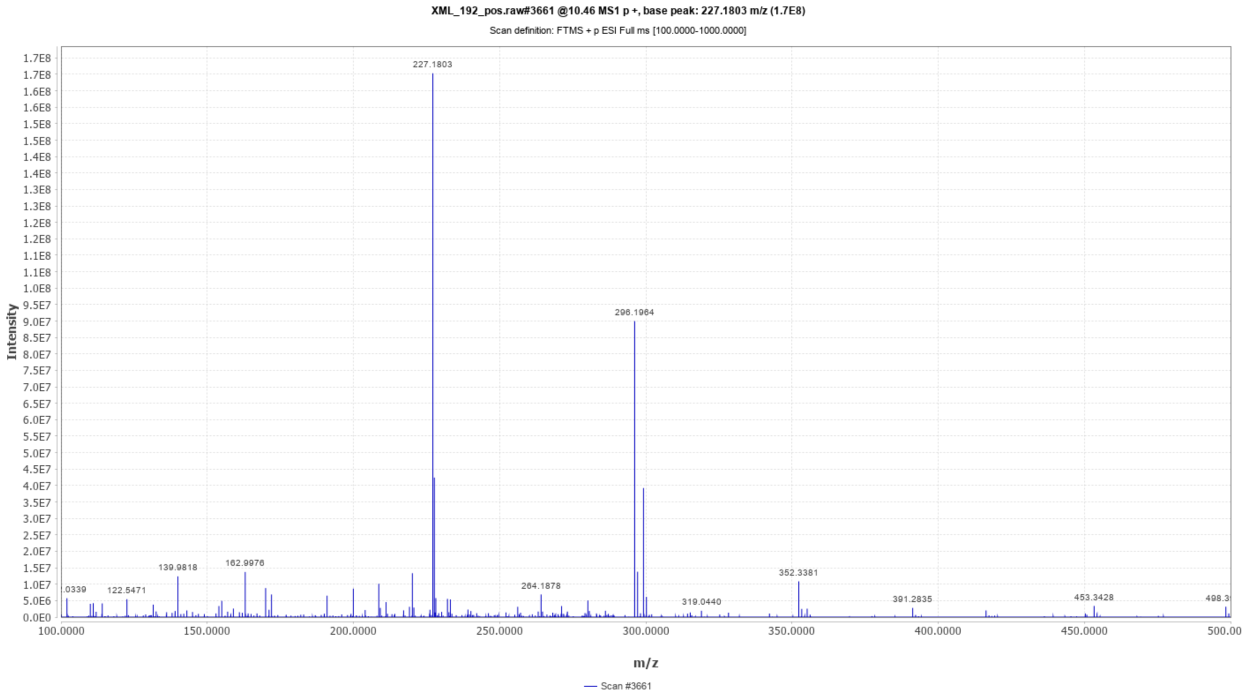

Table S5. Proposed structure of compound **5**

m/z	Rt	Charge state, *z*	M_w exp.*	Proposed Formula*	Δ(ppm) *	Proposed Structure [M+2H]$^{2+}$
227.1803	10.46	2	452.3450	$C_{23}H_{44}N_6O_3$	-3.32	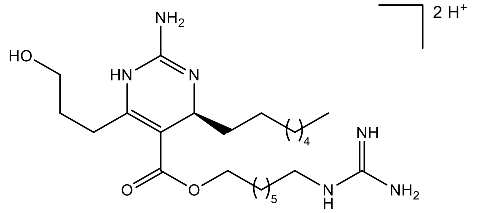

* *corresponding to the non-charged species, calculation of the experimental* M_w *and mass error are detailed in the note to* ***Table 2***.

Figure S6. MS/MS spectrum of compound **5**

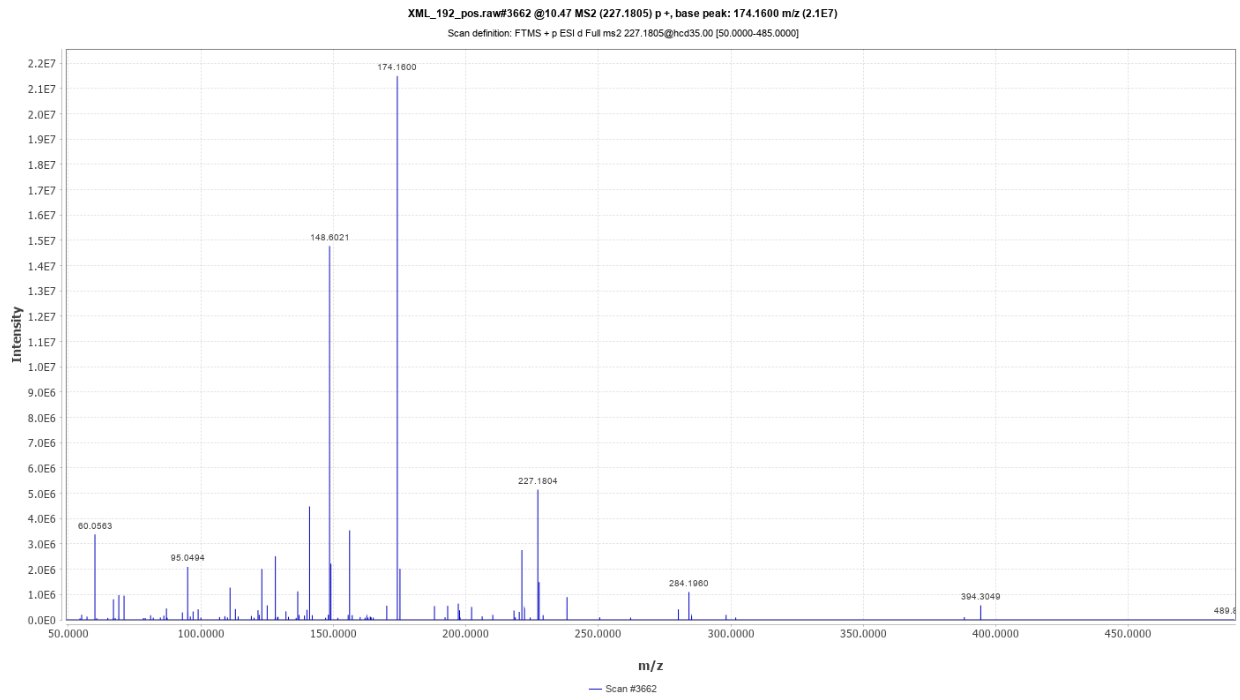

Table S6. Fragmentation of compound **5**

MS/MS fragment m/z	Charge state, *z*	Proposed Formula	Δ(ppm)	Proposed Structure
174.1600	1	$C_8H_{20}N_3O^+$	-1.15	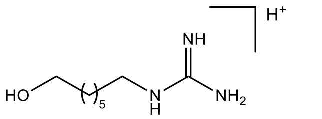
148.6021	2	$C_{15}H_{27}N_3O_3^{2+}$	-0.67	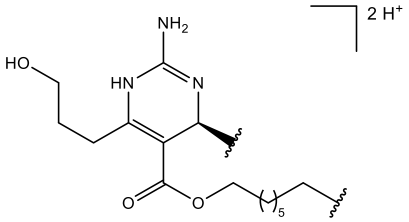

Figure S7. MS spectrum of Crambescin C 466 homologue (m=6, n=4), Compound **6**

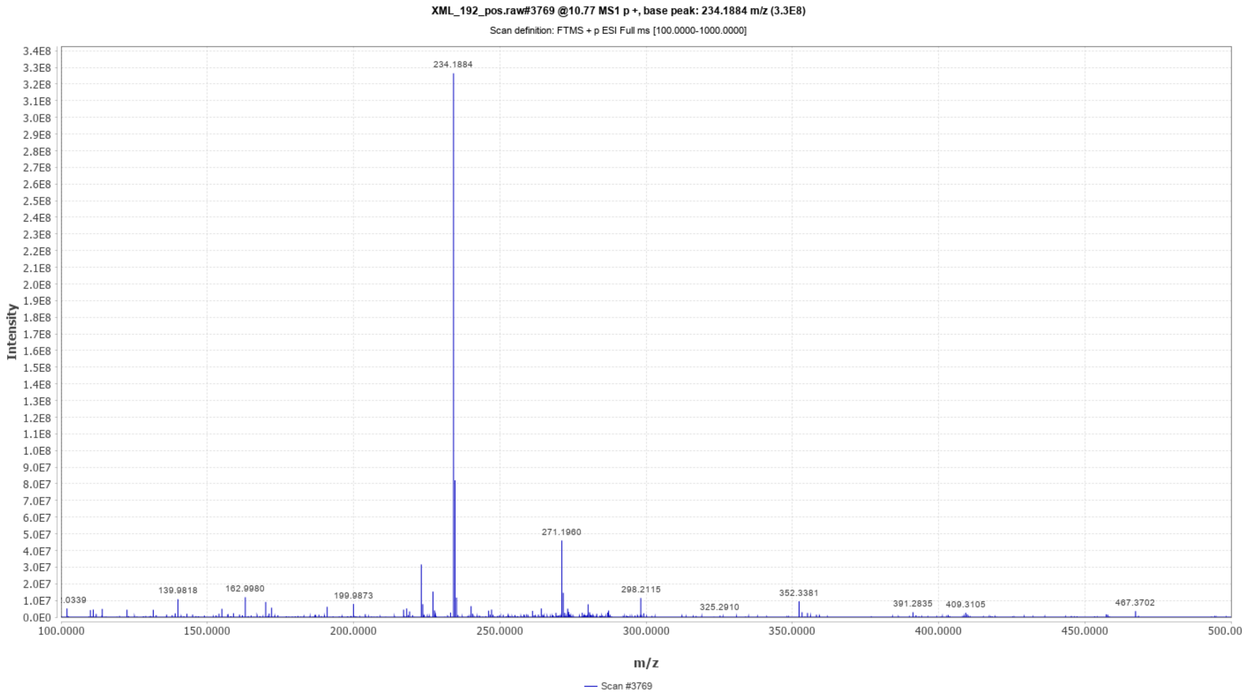

Table S7. Proposed structure of compound **6**

m/z	Rt	Charge state, *z*	M_w exp.*	Proposed Formula*	Δ(ppm) *	Proposed Structure [M+2H]$^{2+}$
234.1884	10.77	2	466.3612	$C_{24}H_{46}N_6O_3$	-1.93	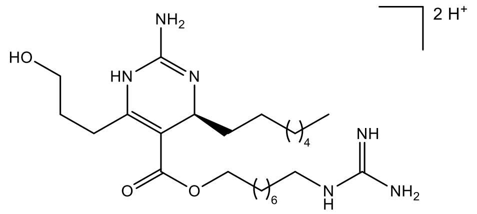

* *corresponding to the non-charged species, calculation of the experimental M_w and mass error are detailed in the note to **Table 2**.*

Figure S8. MS/MS spectrum of compound **6**

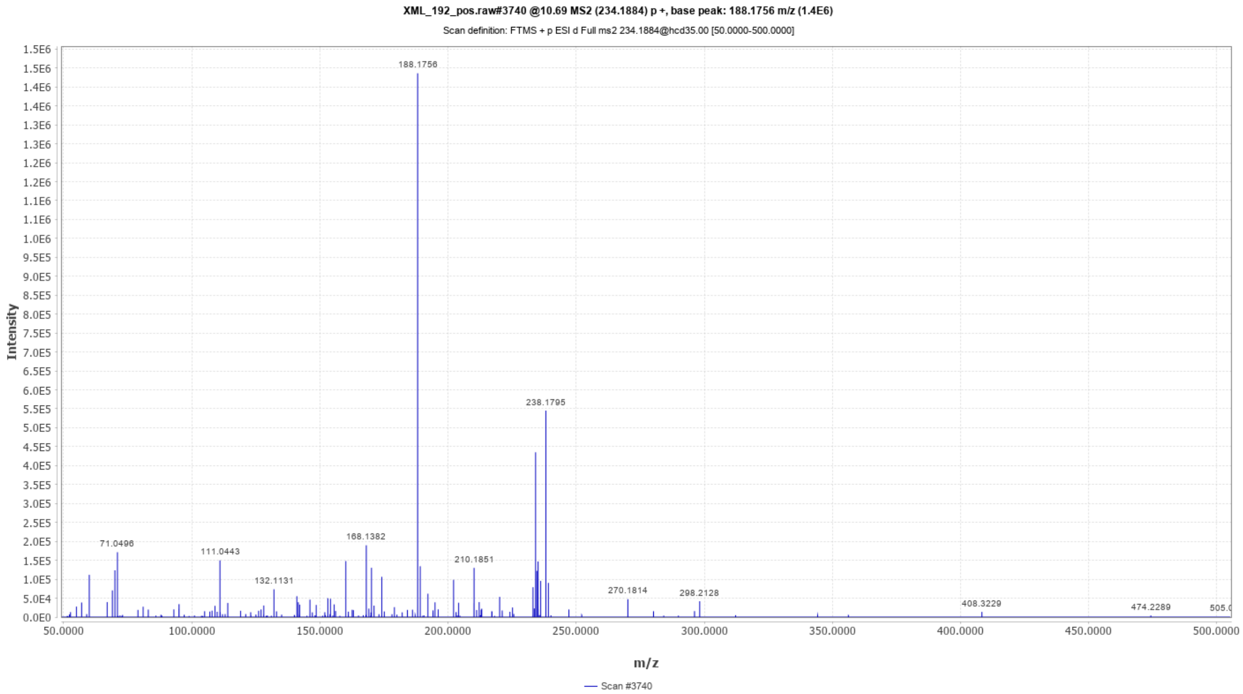

Table S8. Fragmentation of compound **6**

MS/MS fragment m/z	Charge state, *z*	Proposed Formula	Δ(ppm)	Proposed Structure
188.1756	1	$C_9H_{22}N_3O^+$	-1.06	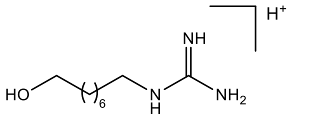
155.6100	2	$C_{16}H_{29}N_3O_3^{2+}$	-0.32	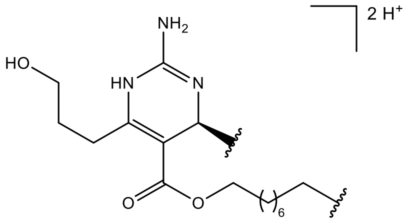

Figure S9. MS spectrum of Crambescin C 466 homologue (m=5, n=5), Compound **7**

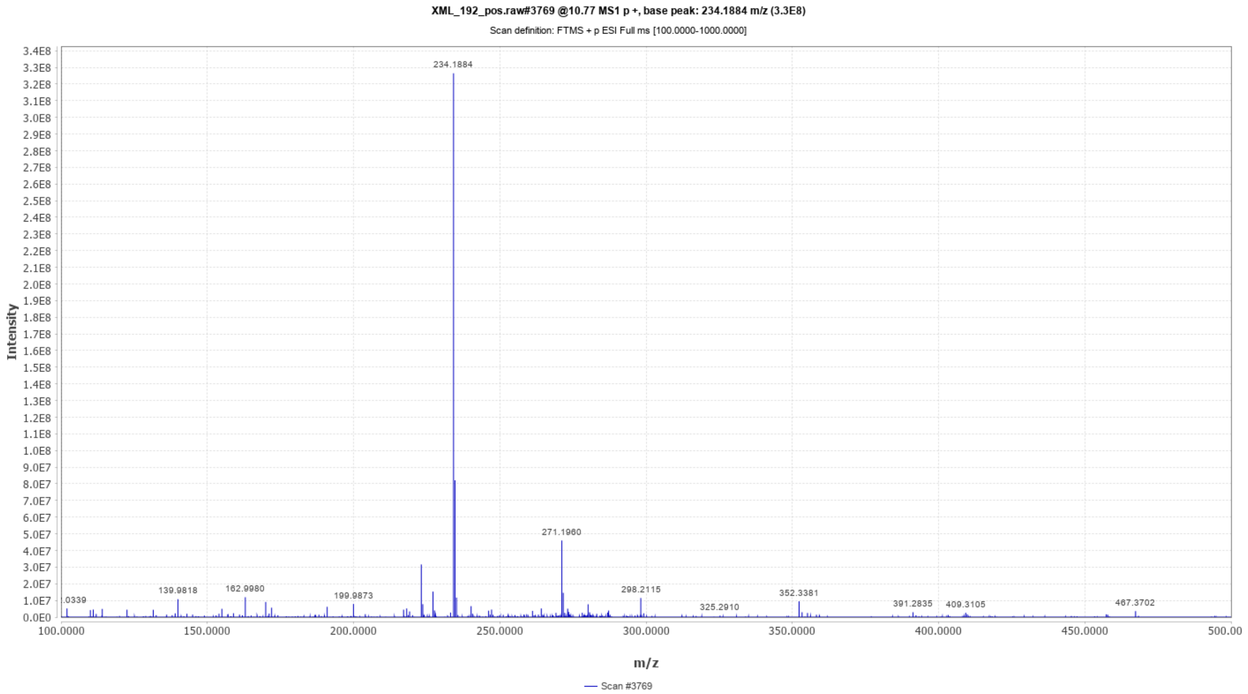

Table S9. Proposed structure of compound **7**

m/z	Rt	Charge state, *z*	Mw exp.*	Proposed Formula*	Δ(ppm) *	Proposed Structure [M+2H]$^{2+}$
234.1884	10.77	2	466.3612	$C_{24}H_{46}N_6O_3$	-1.93	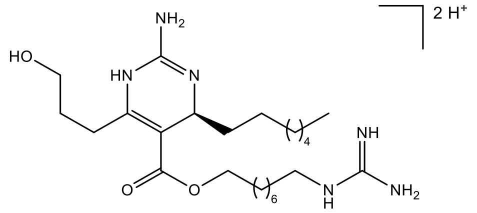

* *corresponding to the non-charged species, calculation of the experimental M_w and mass error are detailed in the note to* ***Table 2***.

Figure S10. MS/MS spectrum of compound **7**

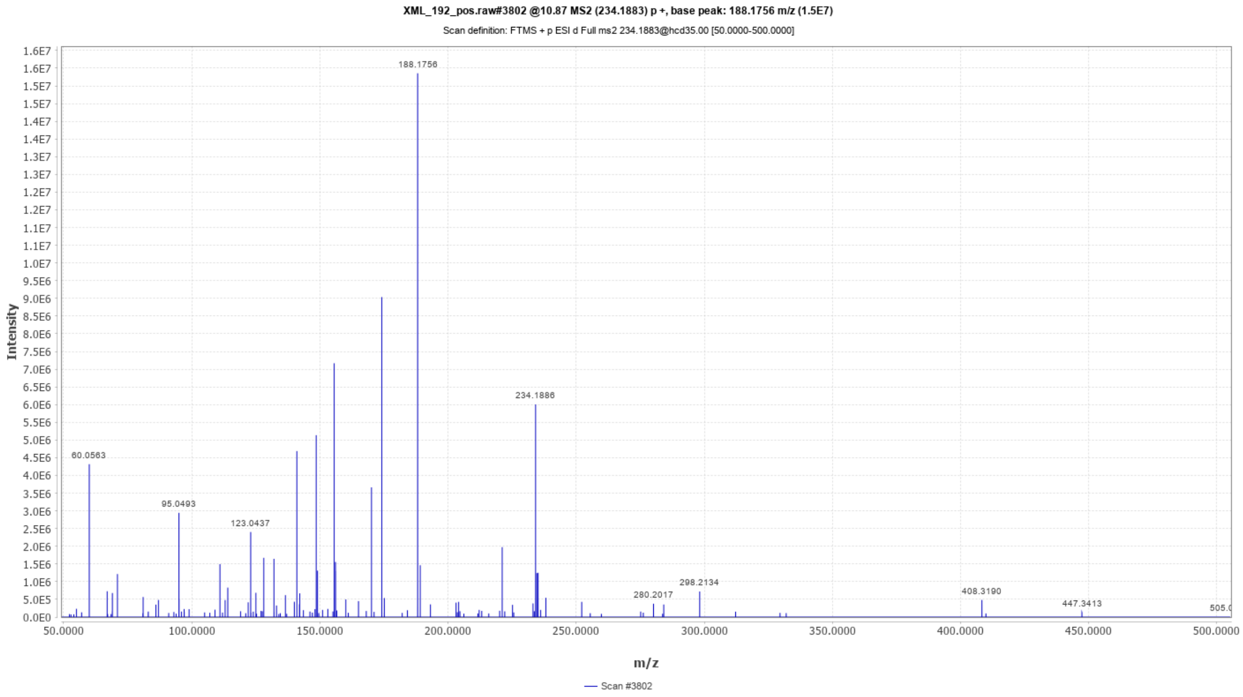

Zoomed in m/z range 50-200:

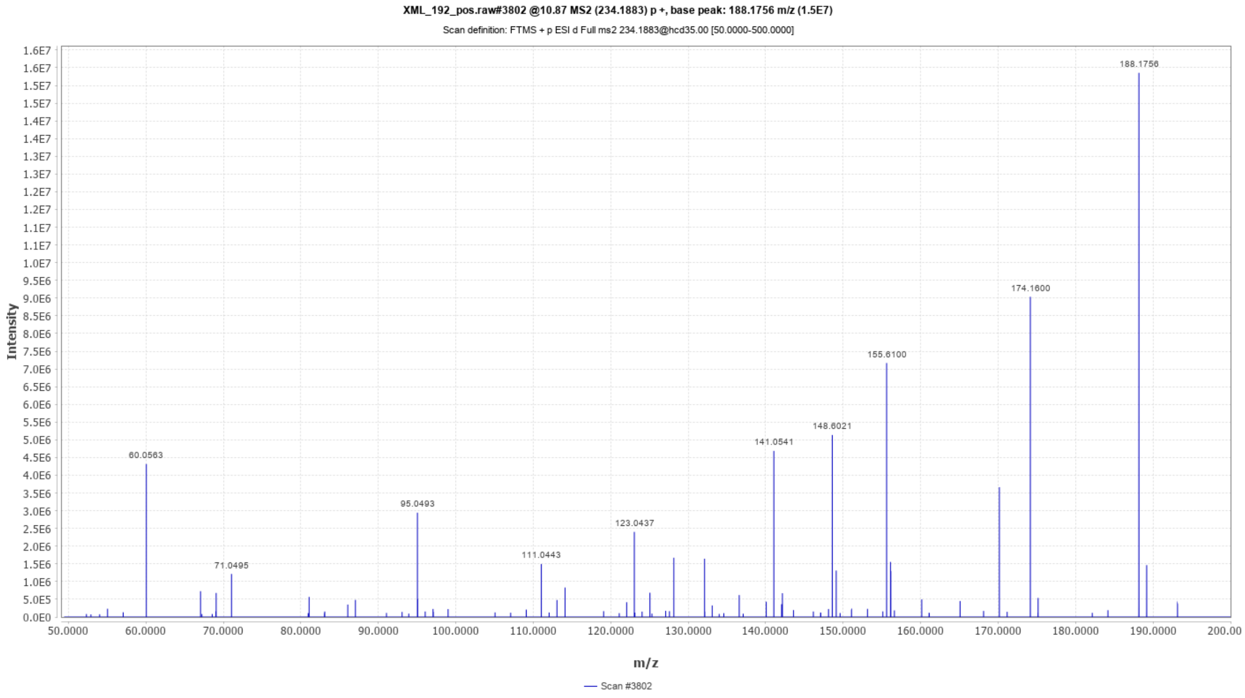

Table S10. Fragmentation of compound **7**

MS/MS fragment m/z	**Charge state, *z***	**Proposed Formula**	**Δ(ppm)**	**Proposed Structure**
174.1600	1	$C_8H_{20}N_3O^+$	-1.15	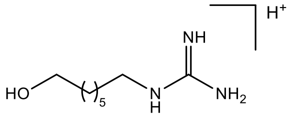
148.6021	2	$C_{15}H_{27}N_3O_3^{2+}$	-0.67	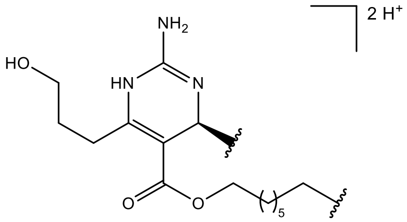

Table S11. Structures of 53 annotated crambescin-, crabescidin- and other guanidine-related compounds

#	Proposed Formula	Proposed identification	Proposed structure
1	$C_{14}H_{23}N_3O_3$	guanidine related compound ($C_{14}H_{23}N_3O_3$)	N/A
2	$C_{14}H_{23}N_3O_3$	guanidine related compound ($C_{14}H_{23}N_3O_3$)	N/A
3	$C_{14}H_{21}N_3O_2$	guanidine related compound ($C_{14}H_{21}N_3O_2$)	N/A
4	$C_{15}H_{25}N_3O_3$	guanidine related compound ($C_{15}H_{25}N_3O_3$)	N/A
5	$C_{23}H_{44}N_6O_3$	crambescin C 452 homologue (m=5, n=4)	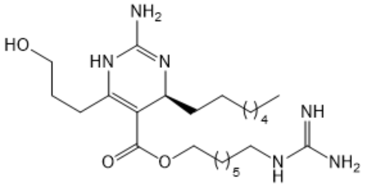
6	$C_{24}H_{46}N_6O_3$	crambescin C 466 homologue (m=6, n=4)	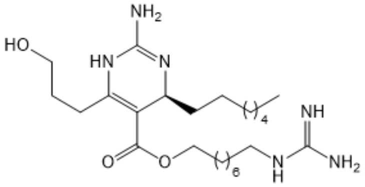
7		crambescin C 466 homologue (m=5, n=5)	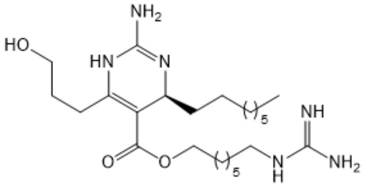
8	$C_{23}H_{42}N_6O_2$	crambescin A 434 homologue (m=5, n=4)	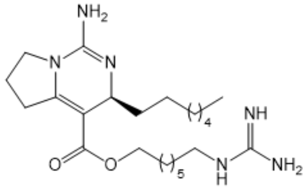
9	$C_{23}H_{44}N_6O_3$	crambescin B 452 homologue (m=5, n=4)	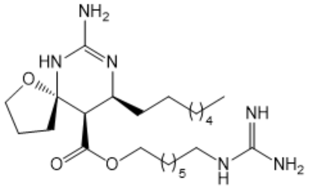
10	$C_{24}H_{46}N_6O_3$	crambescin C1 466 (m=4, n=6)	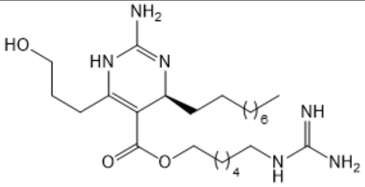

#	Proposed Formula	Proposed identification	Proposed structure
11	$C_{25}H_{48}N_6O_3$	crambescin C 480 homologue (m=6, n=5)	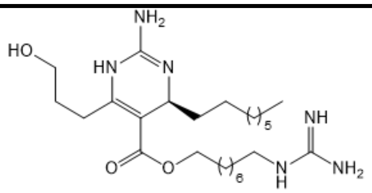
12	$C_{24}H_{44}N_6O_2$	crambescin A 448 homologue (m=6, n=4)	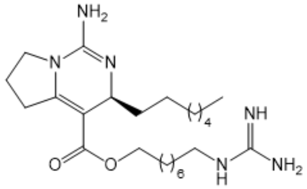
13	$C_{44}H_{87}N_6O_{11}$	crambescidin 875	N/A
14	$C_{24}H_{46}N_6O_3$	crambescin B 466 homologue (m=6, n=4)	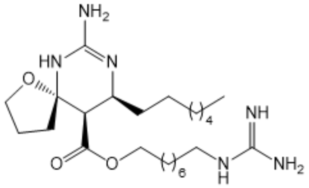
15	$C_{45}H_{82}N_6O_8$	crambescidin 834	N/A
16	$C_{25}H_{48}N_6O_3$	crambescin C1 480 (m=5, n=6)	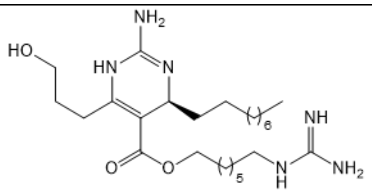
17	$C_{23}H_{42}N_6O_2$	crambescin A2 434 (m=2, n=7)	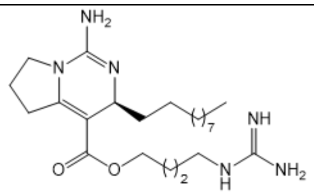
18	$C_{28}H_{44}N_6O_2$	crambescin A3 496 (m=2) (cis)	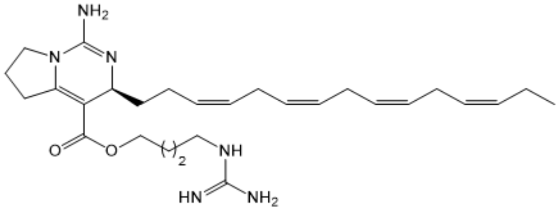
19	$C_{24}H_{44}N_6O_2$	crambescin A1 448 (m=4, n=6)	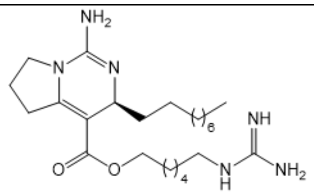

#	Proposed Formula	Proposed identification	Proposed structure
20	$C_{24}H_{46}N_6O_3$	crambescin C2 466 (m=2, n=8)	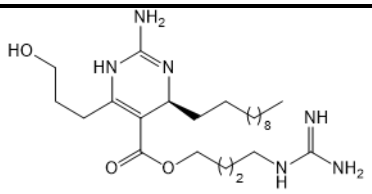
21		crambescin B1 466 (m=4, n=6)	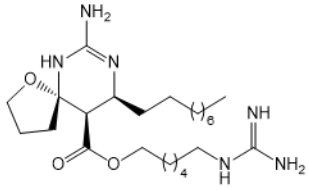
22	$C_{26}H_{50}N_6O_3$	crambescin C1 494 (m=6, n=6)	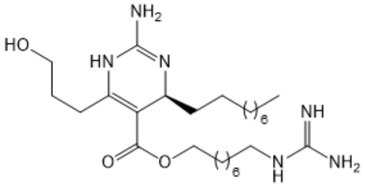
23	$C_{26}H_{46}N_6O_2$	didehydrocrambescin A1 474 (m=6, n=6)	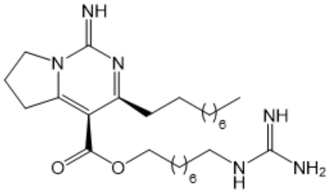
24	$C_{25}H_{48}N_6O_3$	crambescin B 480 homologue (m=6, n=5)	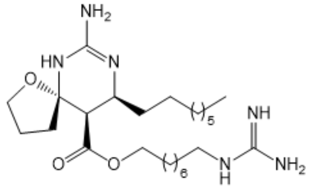
25	$C_{29}H_{46}N_6O_2$	crambescin A3 510 (m=3) (cis)	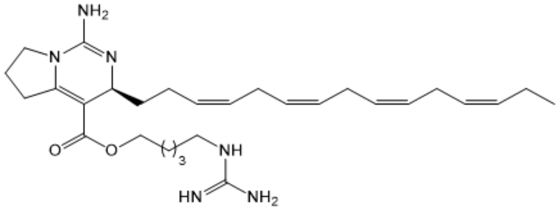
26	$C_{28}H_{44}N_6O_2$	crambescin A3 496 (m=2) (trans)	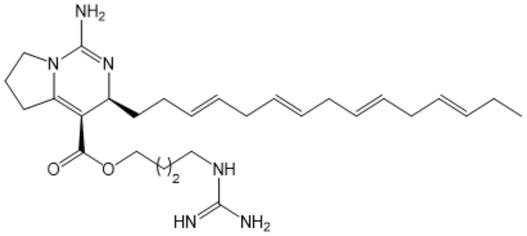
27	$C_{25}H_{46}N_6O_2$	crambescin A1 462 (m=5, n=6)	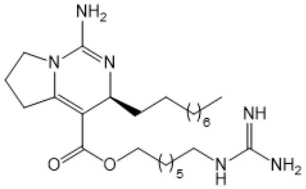

#	Proposed Formula	Proposed identification	Proposed structure
28	$C_{25}H_{48}N_6O_3$	crambescin B1 480 (m=5, n=6)	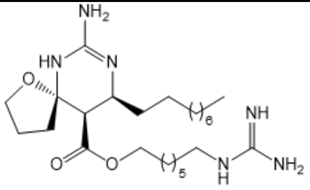
29	$C_{26}H_{50}N_6O_3$	crambescin C2 494 (m=2, n=10)	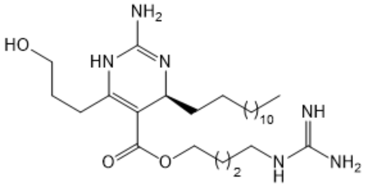
30	$C_{24}H_{44}N_6O_2$	crambescin A2 448 (m=2, n=8)	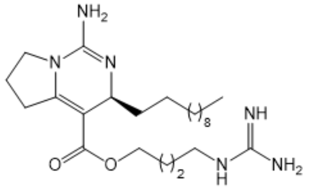
31	$C_{30}H_{50}N_6O_3$	crambescin C3 542(m=4)	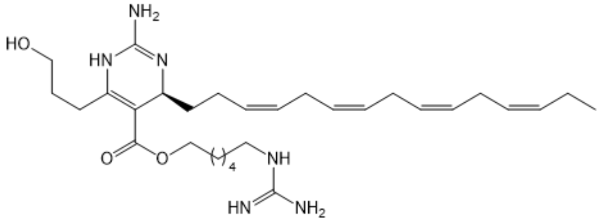
32	$C_{45}H_{80}N_6O_6$	crambescidin 800 or isocrambescidin 800	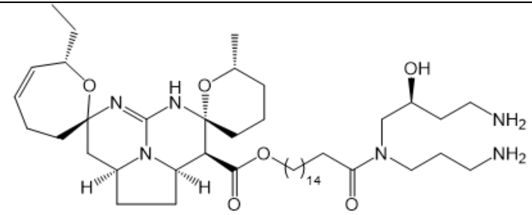 or 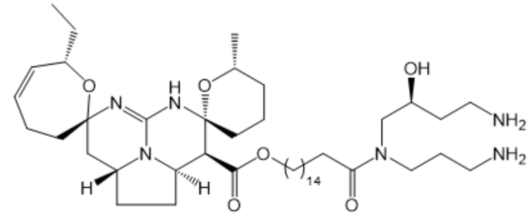
33	$C_{30}H_{48}N_6O_2$	crambescin A3 524 (m=4) (*cis*)	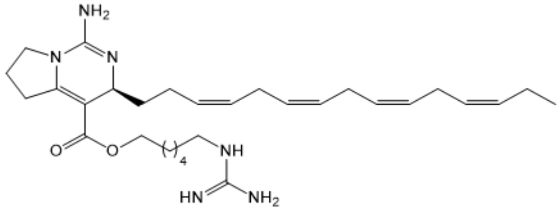

#	Proposed Formula	Proposed identification	Proposed structure
34	$C_{29}H_{46}N_6O_2$	crambescin A3 510 (m=3) (*trans*)	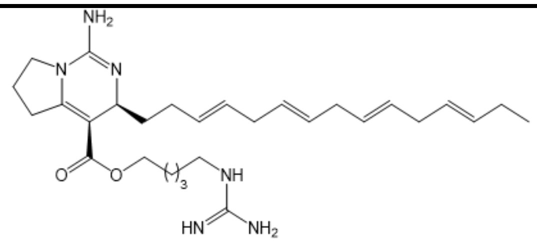
35	$C_{44}H_{85}N_6O_{10}$	crambescidin 857	N/A
36	$C_{45}H_{80}N_6O_7$	crambescidin 816	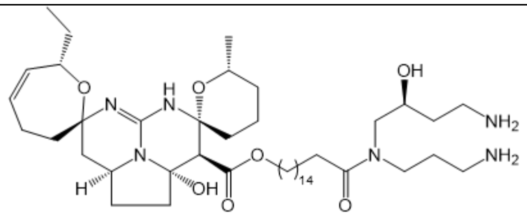
37	$C_{24}H_{46}N_6O_3$	crambescin B2 466 (m=2, n=8)	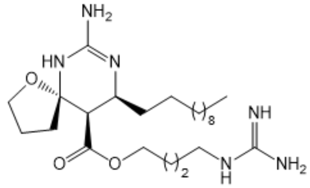
38	$C_{26}H_{48}N_6O_2$	crambescin A1 476 (m=6, n=6)	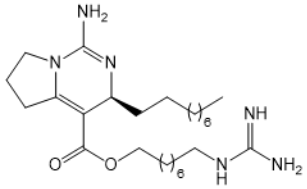
39	$C_{44}H_{85}N_6O_9$	crambescidin 841	N/A
40	$C_{45}H_{80}N_6O_6$	crambescidin 800 or isocrambescidin 800	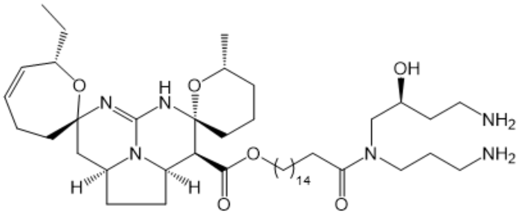 or 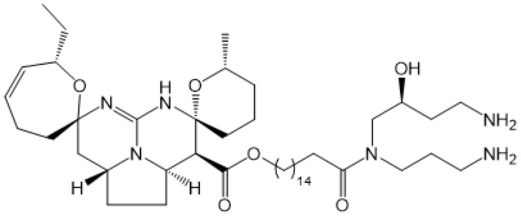
41	$C_{26}H_{50}N_6O_3$	crambescin B1 494 (m=6, n=6)	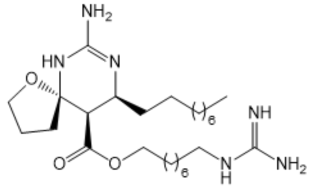

#	Proposed Formula	Proposed identification	Proposed structure
42	$C_{25}H_{46}N_6O_2$	crambescin A 462 homologue (m=3, n=8)	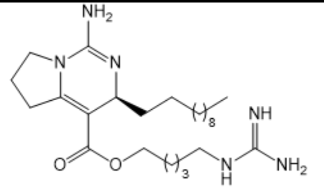
43	$C_{26}H_{46}N_6O_2$	didehydrocrambescin A2 474 (m=2, n=10)	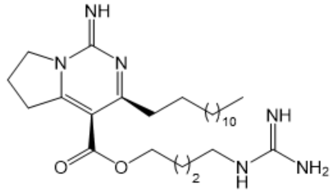
44	$C_{31}H_{50}N_6O_2$	crambescin A3 538 (m=5) (*cis*)	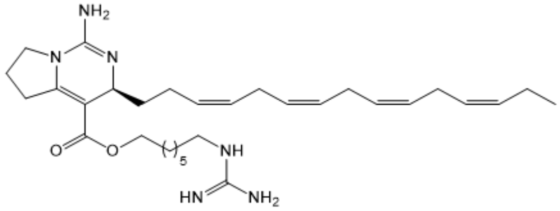
45	$C_{30}H_{48}N_6O_2$	crambescin A3 524 (m=4) (*trans*)	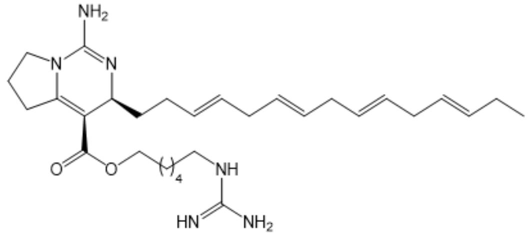
46	$C_{25}H_{46}N_6O_2$	crambescin A2 462 (m=2, n=9)	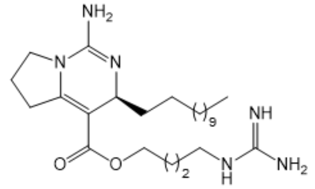
47	$C_{46}H_{82}N_6O_7$	crambescidin 830	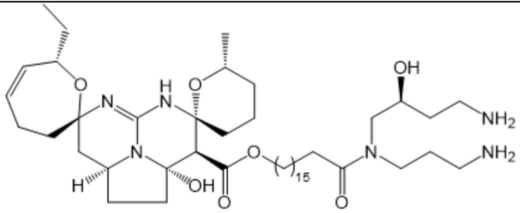
48	$C_{30}H_{50}N_6O_3$	crambescin B3 542 (m=4)	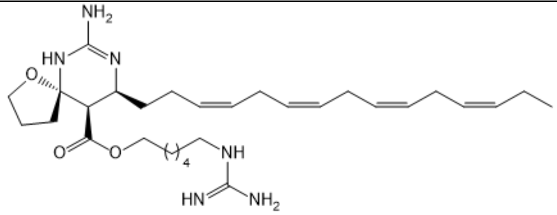

#	Proposed Formula	Proposed identification	Proposed structure
49	$C_{31}H_{50}N_6O_2$	crambescin A3 538 (m=5) (trans)	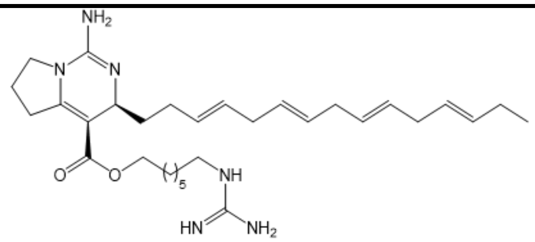
50	$C_{22}H_{33}O_4N_3$	crambescidin acid	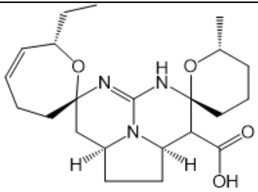
51	$C_{26}H_{48}N_6O_2$	crambescin A2 476 (m=2, n=10)	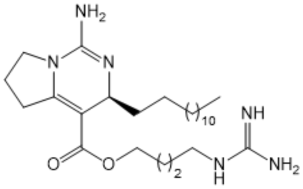
52	$C_{14}H_{27}N_3O$	crambescin 253	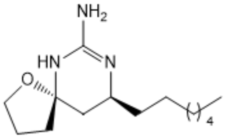
53	$C_{16}H_{31}N_3O$	crambescin 281	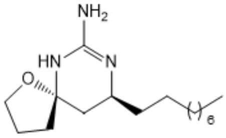
